# Detection of analytes in mitochondria without interference from other sites based on an innovative ratiometric fluorophore†
†Electronic supplementary information (ESI) available. See DOI: 10.1039/c8sc01673a


**DOI:** 10.1039/c8sc01673a

**Published:** 2018-05-22

**Authors:** Tian-Bing Ren, Qian-Ling Zhang, Dongdong Su, Xing-Xing Zhang, Lin Yuan, Xiao-Bing Zhang

**Affiliations:** a State Key Laboratory of Chemo/Biosensing and Chemometrics , College of Chemistry and Chemical Engineering , Hunan University , Changsha 410082 , PR China . Email: lyuan@hnu.edu.cn; b College of Chemistry and Chemical Engineering , Tianjin University of Technology , Tianjin 300384 , PR China

## Abstract

Mitochondria are vital organelles that not only produce cellular energy but also participate in many biological processes. Recently, various fluorescent probes have been developed for mitochondrial imaging. However, due to the lack of suitable dyes or strategies, it is difficult for most reported mitochondrial targeting probes to prove whether the analytes they detected are from mitochondria. In addition, positive charge on mitochondrial probes can seriously affect the mitochondrial environment. To address these issues, we herein put forward a novel strategy for probe design based on a smart NIR dye (**HDFL**) for mitochondrial targeting detection. Compared to general mitochondrial targeting probes that are modified with a target site and a reaction site, the new strategy is to combine the two sites together for a mitochondrial probe that would provide accurate detection of analytes in mitochondria without interference. As a proof of concept, we synthesized a mitochondrial-targetable probe **HDFL-Cys** for cysteine. Bioimaging studies have shown that the new type of probe **HDFL-Cys** can first accumulate in mitochondria and then react with the analyte (cysteine) accompanied by the departure of the targeting group (lipophilic cation moieties). Thus, it can specifically detect the analyte in mitochondria without interference from extra-mitochondrial analytes. We anticipate that the new strategy based on the novel NIR dye **HDFL** may be a potential platform for developing desirable ratiometric fluorescent probes for mitochondrial imaging.

## Abstract 2

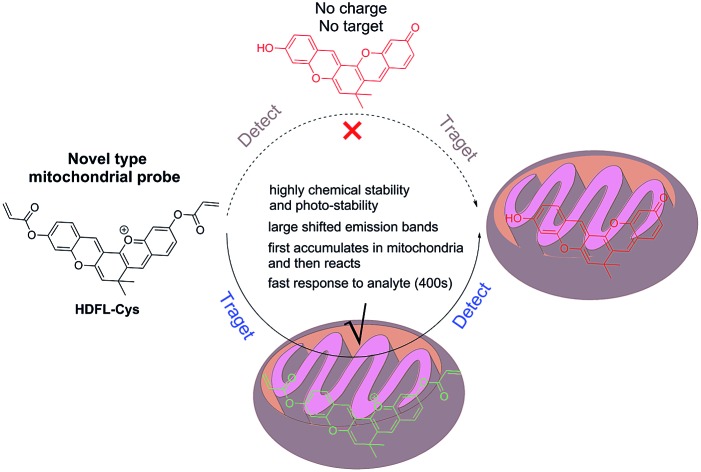
A new strategy that integrates the targeting group and response moiety together for the preparation of mitochondrial probe was developed. Bioimaging studies have shown that for the first time, the newly designed probe **HDFL-Cys** can first accumulate in mitochondria and then react with the analyte.

## Introduction

**Fig. 1:**
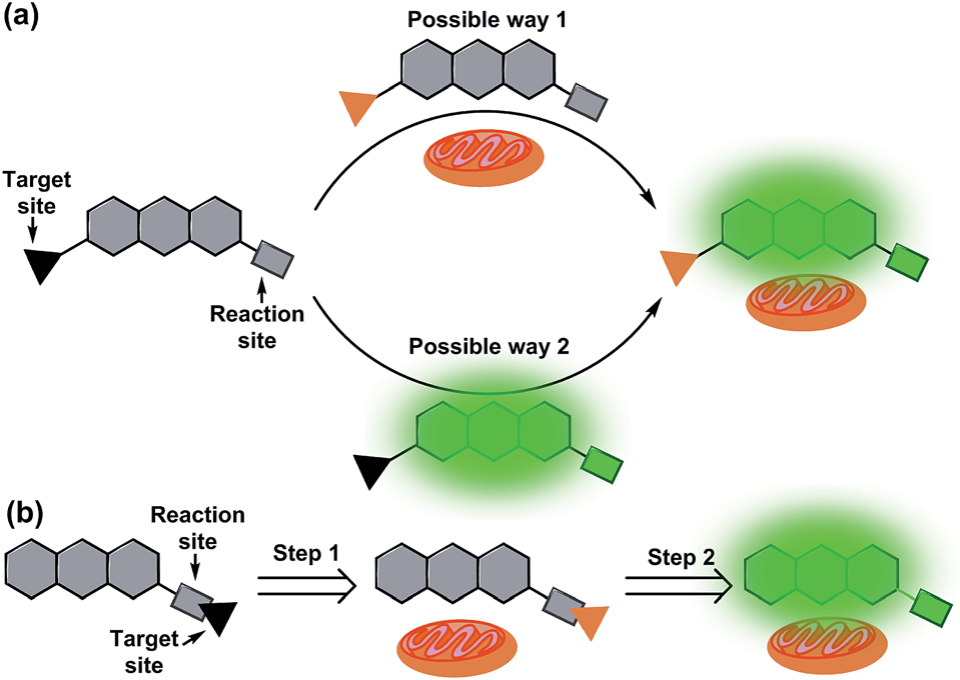
Illustration of a general mitochondrial probe (a) and the novel mitochondrial probe (b), and their application in mitochondrial imaging.

Mitochondria, a kind of vital organelle, exist in most eukaryotic cells, and not only produce cellular energy, but also participate in many biological processes such as signalling, and cell differentiation, growth, and death.^1^–^3^ As a highly delicate system, mitochondria require punctual and well-matched signalling elements to trigger each step of the reactions.^3^ Prominent signal molecules include various metal ions^4^–^7^ and reactive oxygen/nitrogen/sulfur species (ROS/RNS/RSS).^8^–^14^ In order to better understand the roles and functions of these analytes in mitochondria, various mitochondrial targeted fluorescent probes have recently been developed.^3^,^15^–^18^ Because of the strong negative membrane potential in the matrix of mitochondria (as high as 180–200 mV),^19^,^20^ in general, lipophilic cations have a high tendency to localize in mitochondria. Therefore, most mitochondrial targeted fluorescent probes are developed with a lipophilic cationic moiety.^21^–^26^ However, accurate positioning as the lipophilic cationic is, these mitochondrial probes still suffer from a series of practical issues. For example, most of these mitochondrial probes are positively charged before and after the response to the analytes. But what's baffling is whether the probes accumulate first in mitochondria and then react with analytes or if they react with analytes before accumulating in mitochondria (Fig. 1a). Meanwhile, due to the positive charge of the probes, the membrane potential of mitochondria may also be affected, resulting in membrane rupture and disturbance of the microenvironment.^27^–^30^ To solve these problems, further optimization of the mitochondrial-targeting strategy is critical.

Ideally, fluorescent probes suitable for imaging mitochondria should first accumulate in the mitochondria and then react with the analytes. However, due to the lack of corresponding dyes and strategies, most reported mitochondrial targeted probes cannot realize and visualize this process. In addition, it is also very important that mitochondrial probes should not disturb the microenvironment, especially the membrane potential of mitochondria. This means that the mitochondrial probes preferably remain neutral or become neutral after entering the mitochondria. With this in mind, herein, we put forward a novel strategy for the design of mitochondrial-targeting detection probes. As shown in Fig. 1b, different from conventional methods for developing mitochondrial-targeting probes, we proposed a new strategy that integrates the target site and reaction site together for the preparation of mitochondrial probes. We aim for a novel type of probe that can target mitochondria first, and then detect the analytes in mitochondria accompanied by the departure of the targeting group (lipophilic cation moieties). To achieve this goal and make it visible, we believe that the fluorophore used for designing the probe must be smart enough.

**Fig. 2:**
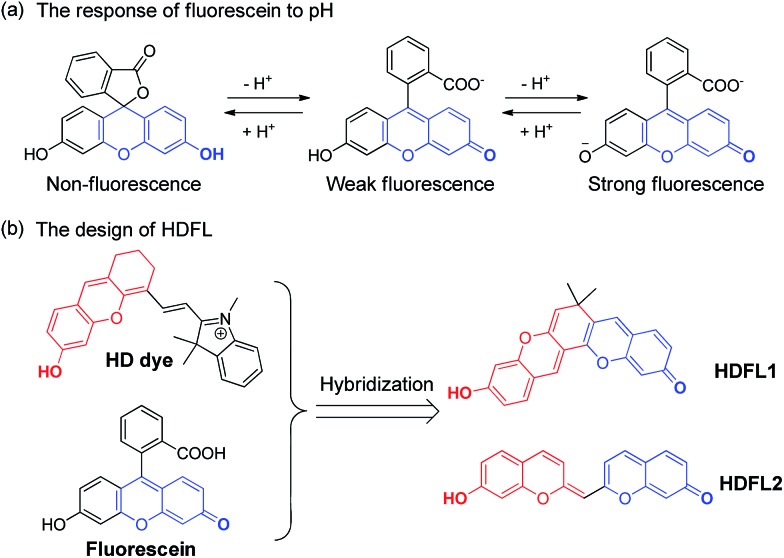
(a) The response of fluorescein to pH. (b) The design of the novel **HDFL** dyes by hybridization of the HD dye and fluorescein.

Herein, inspired by HD dyes and fluorescein, we have developed an innovative NIR dye (**HDFL**, Fig. 2). This new type of fluorophore not only possesses high chemical stability and photostability, together with good biocompatibility for bioimaging, but can also be exploited as a novel platform for designing mitochondrial-targetable ratiometric fluorescent probes with an NIR emission. It is worth noting that the new NIR dyes show a large shifted emission band (>120 nm) by easy modification on the hydroxyl group. Furthermore, as a proof of concept, we have applied the **HDFL** dye to construct a ratiometric fluorescent probe **HDFL-Cys** for cysteine imaging in mitochondria. Bioimaging studies have demonstrated that the probe **HDFL-Cys** can first accumulate in mitochondria and then react with the signalling elements (cysteine). To the best of our knowledge, this is the first mitochondrial targeted probe to demonstrate this. Because of its high sensitivity and selectivity in response to cysteine, **HDFL-Cys** can also be used to distinguish between normal cells and cancer cells by monitoring the cysteine levels. These data lay a firm foundation that our **HDFL** dye can be used as a potential platform for the development of desirable ratiometric fluorescent probes.

## Results and discussion

### Design and synthesis of **HDFL**

Recently, our group has presented a strategy for the construction of merocyanine dyes and HD NIR dyes.^31^,^32^ The dyes possess an optically tunable hydroxyl group and exhibit maximal absorption and emission in the NIR region. However, because of the severe spectral overlap with the hydroxyl decorated in the emission (the spacing of the two emission peaks is fixed at ∼34 nm), HD NIR dyes are difficult to design for ratiometric NIR probes. Comparison of fluorescence changes in one emission band, ratiometric fluorescent probes are more reliable due to their favorable multichannel fluorescence signal output and independence from experimental conditions.^33^–^35^ Therefore, ratiometric fluorescent probes have been widely used for cell or tissue imaging. Structural analysis showed that HD NIR dyes are composed of a 2,3-dihydro-1*H*-xanthene core (donor) connected to an indolium moiety (acceptor). When the electron donating ability of the donor group weakened, the ICT effect of the HD NIR dye may change slightly. Thus, the HD NIR dye exhibited a small shifted emission band. Inspired by fluorescein where the two hydroxyl groups can greatly change its spectral properties (Fig. 2a), we envisioned that hybridization of the fluorescein and HD NIR dye may afford an innovative type of NIR dye, **HDFL**. It is worth noting that **HDFL** contains two critical hydroxyl groups that can greatly change the ICT effect of the HD NIR dye and is suitable for ratiometric imaging (Fig. 2b). The synthesis of the fluorophore **HDFL** was accomplished in only one step, as shown in Scheme S1,† and all of them were carefully characterized by ^1^H NMR, ^13^C NMR and HRMS (ESI†).

### Photophysical properties of fluorescent dyes **HDFL1–2**

**Fig. 3:**
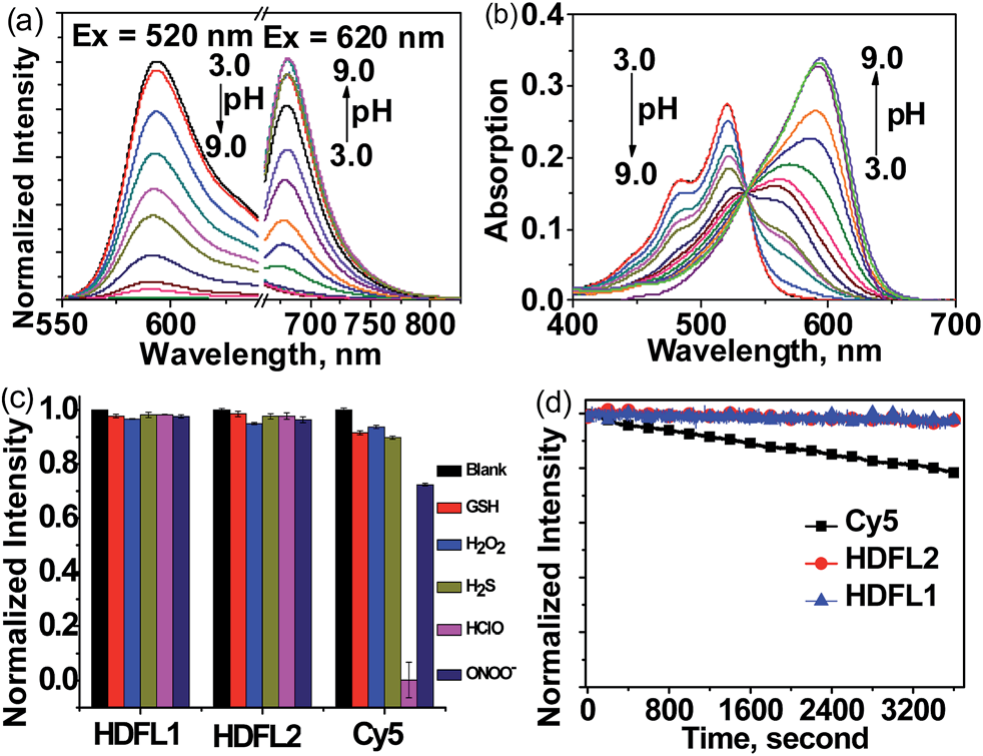
(a) The normalized emission intensity of **HDFL1** in PBS buffer (containing 20% EtOH) with pH changing from 3.0 to 9.0. (b) The absorption spectra of **HDFL1** in PBS buffer (containing 20% EtOH) with pH changing from 3.0 to 9.0. (c) Relative fluorescence intensities of three compounds (5 μM) for various agents in PBS (25 mM, pH 7.4, containing 20% EtOH): H_2_O_2_, 100 μM; H_2_S, 100 μM; HOCl, 100 μM; ONOO^–^, 25 μM; GSH, 1 mM. (d) Time-dependent normalized fluorescence emission intensity of compounds **HDFL1** (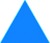), **HDFL2** (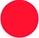), and Cy5 (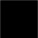) in PBS (25 mM, pH 7.4, containing 20% EtOH) after continuous irradiation for 1 hour.

With **HDFL** dyes in hand, we first tested their optical properties in various solvents (Fig. S1 and 2 and Tables S1 and S3†). Fluorescence spectra showed that both **HDFL1** and **HDFL2** had a larger redshift emission in PBS buffer (pH = 7.4, containing 20% EtOH) than other solvents, with maximum emission peaks at 681 nm (*Φ* = 4.3%) and 684 nm (*Φ* = 3.4%), respectively. Meanwhile, the absorption spectra showed that **HDFL1** and **HDFL2** had broad absorption bands in polar solutions due to their various forms of dissociation and vibrational states (Scheme S2†). Notably, in PBS buffer, **HDFL1** and **HDFL2** exhibited maximum absorption peaks in the far-red region at around 620–650 nm, with substantial molar absorption coefficients (>10^4^). These behaviours of **HDFL1** and **HDFL2** are in good agreement with those of the classic hydroxyl group dyes.^31^,^36^ To get more insight into the influence of hydroxyl groups in **HDFL** dyes, we next examined the pH effect on the photophysical properties of **HDFL1** and **HDFL2** in PBS. The standard pH titrations of **HDFL1** and **HDFL2** (5 μM) were performed in aqueous solution (containing 20% EtOH). As expected, as the pH increased from 3.0 to 9.0, the emission peak of **HDFL1** around 592 nm decreased significantly, while a new red-shifted emission band was observed around 681 nm (Fig. 3a and S3†). This large red-shift (89 nm) in the emission spectra with increased pH can be explained by the deprotonation of **HDFL1**. In addition, by analyzing the emission intensity changes of **HDFL1** as a function of pH by using the Henderson–Hasselbalch-type mass action equation, we acquired p*K*_a_ values of 5.54 and 7.36 for 592 nm and 681 nm, respectively (Fig. S3c and d†).^37^ The results indicate that there are indeed two hydroxyl groups involved in regulating the fluorescence of **HDFL1**, which is in good agreement with our design. An obvious red-shift from 520 to 593 nm in the absorption spectra (Fig. 3b) was also observed due to the deprotonation of **HDFL1**. The pH dependent absorption/emission spectra of **HDFL2** showed a similar trend to those of **HDFL1** with the increase of pH from 3.0 to 11.0 (Fig. S4–6†). Therefore, we conclude that the novel **HDFL** dyes can be exploited as a novel platform for designing ratiometric fluorescent probes by the modification on the hydroxyl group.

To confirm the bioavailability of **HDFL** dyes, we then examined their chemical stability and photo-stability under physiological conditions. Their chemical stability toward a range of oxidants and reductants, including common reactive oxygen species (HClO, H_2_O_2_, and ONOO^–^), bio-relevant nucleophiles and reducing agents (GSH and H_2_S), was evaluated by emission spectral analysis in aqueous solution. The results were compared with those of Cy5 under the same conditions. As shown in Fig. 3c and S7–11,† the fluorescence intensity of **HDFL1** remained constant when various oxidants and reductants were added to the PBS solution for 1 hour, whereas Cy5 had an obvious fluorescence loss under the same conditions, especially when HClO was added, the fluorescence was lost almost completely. The fluorescence intensity of **HDFL2** also remained stable in the presence of various oxidants and reductants (Fig. S12–16†). To test their photo-stability, solutions of **HDFL1**, **HDFL2** and Cy5 in PBS (7.4) were continuously irradiated by means of a Xe-lamp at their maximal absorption wavelengths. It was observed that both **HDFL1** and **HDFL2** were stable after 1 hour of continuous irradiation (>97% of the initial values), while the fluorescence of commercial Cy5 decreased to approximately 78% of the initial value under the same irradiation conditions (Fig. 3d). Similar results were also observed in the living cell experiments (Fig. S21†). All these data demonstrate that **HDFL** dyes are more photo-stable than Cy5. The high chemical stability and photo-stability of **HDFL** dyes indicate that they can be used as excellent organic fluorophores for imaging applications.

### Synthesis and sensing properties of **HDFL-Cys** to cysteine

**Fig. 4:**
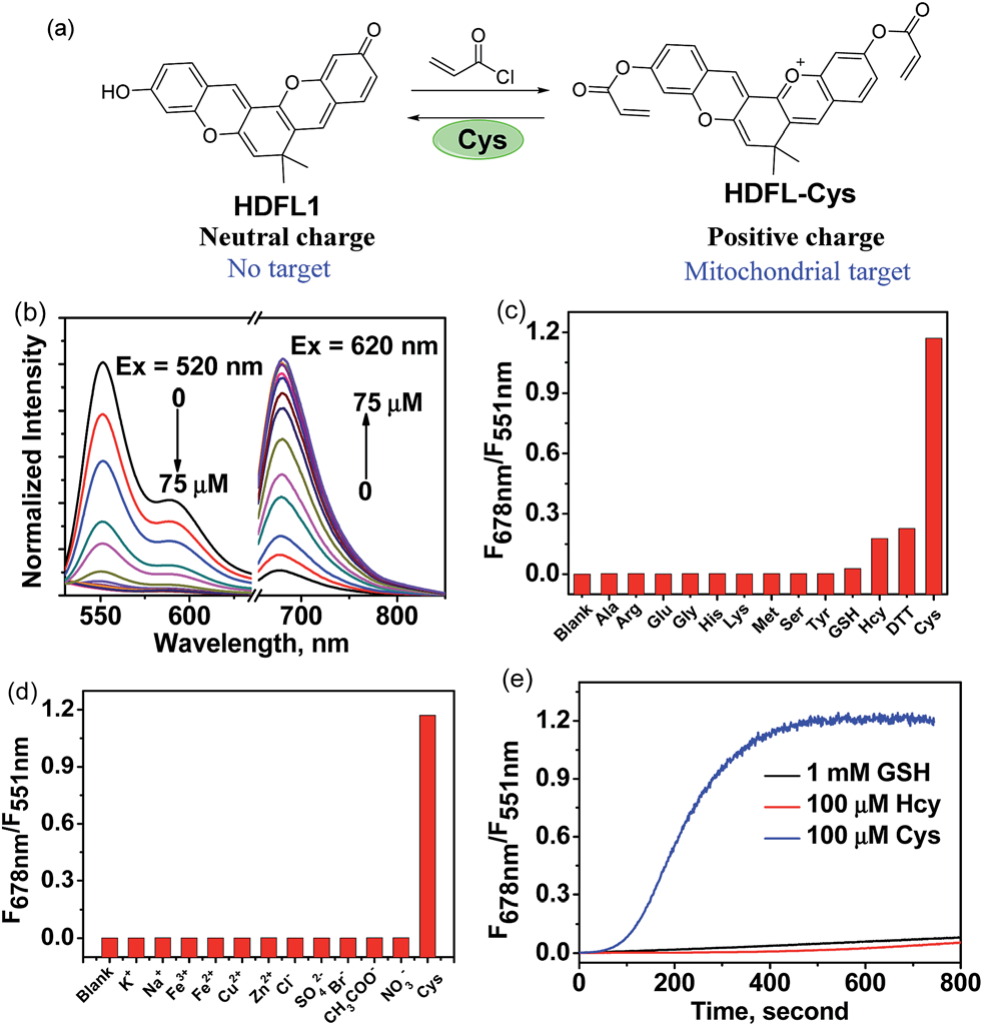
(a) Design and synthesis of probe **HDFL-Cys** for Cys. (b) Normalized fluorescence spectra of **HDFL-Cys** (5 μM) in the presence of various concentrations of Cys (0–75 μM) in PBS buffer (25 mM, pH 7.4, containing 20% EtOH) under excitation at 520 nm and 620 nm. (c) Fluorescence spectra of **HDFL-Cys** (5 μM) in the presence of Cys (100 μM), other biological thiols or amino acids (100 μM) in PBS buffer (25 mM, pH 7.4, containing 20% EtOH). (d) Fluorescence spectra of **HDFL-Cys** (5 μM) in the presence of Cys (100 μM) and other ions (100 μM) in PBS buffer (25 mM, pH 7.4, containing 20% EtOH). (e) Time-dependent emission ratio change of **HDFL-Cys** (5 μM) upon addition of Cys (100 μM), Hcy (100 μM), and GSH (1 mM).

Since Strongin *et al.* first reported the acrylate moiety,^38^ it has already become a good reaction site for the detection of cysteine over Hcy and GSH *via* the addition–cyclization reaction mechanism.^39^–^41^ Particularly, double acrylate-functionalized probes can increase the selectivity of the probe for Cys, because each biothiol has a different reaction kinetics.^42^ To verify the possibility that **HDFL** dyes can be used for *in vivo* detection and imaging, here, we developed probe **HDFL-Cys** (Fig. 4a) as a novel candidate by facilely modifying the hydroxyl group of **HDFL1** with two acrylate moieties. On the other hand, **HDFL1** with two acrylate moieties decorated, a neutral compound with no target, could become a positively charged compound (**HDFL-Cys**), which has a high tendency to localize in mitochondria. The novel type probe **HDFL-Cys** is expected to recognize cysteine in mitochondria with non-interference of the microenvironment.

With the **HDFL-Cys** probe, we first evaluated its optical response to cysteine. As shown in Fig. 4b and S17,† the free **HDFL-Cys** displayed a yellow emission band centred at 551 nm (*Φ* = 45.8%, Table S4†). However, with the addition of cysteine (0–75 μM), the yellow emission band at 551 nm gradually disappeared, and the NIR emission band with a peak at 678 nm increased (*Φ* = 4.3%, Table S3†). The dramatic change with a large shifted emission band (127 nm) is beneficial for accurate ratiometric imaging.^43^,^44^ In addition, the fluorescence intensity ratio at 678 nm and 551 nm (*F*_678_/*F*_551_) was increased by 1847-fold (Fig. 4c) and showed a linear response to cysteine with a detection limit of 0.8 ± 0.02 nM [(3 × standard deviation)/slope]^45^ (Fig. S18†). pH influence on the **HDFL-Cys** probe response was also studied. As shown in Fig. S19,† the fluorescence intensity ratio of the free probe **HDFL-Cys** was not influenced by a wide range of pH from 5.0 to 8.5, indicating that the free probe was stable in the physiological environment. With cysteine added, **HDFL-Cys** exhibited a substantial response at pH 7.0–8.5, implying that the probe is suitable for Cys detection at the physiological pH of 7.4. To investigate the selectivity, **HDFL-Cys** (5 μM) was then treated with various interfering species, including essential amino acids (Ala, Arg, Glu, Gly, His, Lys, Met, Ser, and Tyr), biothiols (Cys, Hcy, GSH, and DTT), anions (Cl^–^, SO_4_^2–^, Br^–^, AcO^–^, and NO_3_^–^) and cations (K^+^, Na^+^, Fe^2+^, Fe^3+^, Cu^2+^, and Zn^2+^). As shown in Fig. 4c and d, **HDFL-Cys** exhibits a much higher response to cysteine over other biological species. Time-course studies showed that **HDFL-Cys** has a very rapid ratiometric response to cysteine (100 μM), achieving the maximum ratio within 400 seconds, while GSH and Hcy showed only a very weak response with time extending to 800 seconds (Fig. 4e). This response time is much shorter than those of the known cysteine probes (Table S5†). All these results indicated that **HDFL-Cys** had an excellent sensitivity and selectivity to cysteine, and was potentially useful for fast ratiometric detection of cysteine in living cells.

### Reaction mechanism study

To investigate the reaction mechanism of **HDFL-Cys**, mass spectrometry of **HDFL-Cys** and cysteine was then performed. As shown in Fig. S20,† after addition of cysteine, a peak at *m*/*z* 345.0 [M + H^+^] was observed, consistent with that of the expected product, **HDFL1**. The course of the reaction can reasonably explain the optical response of **HDFL-Cys** to Cys and also agrees with the reports.^38^–^41^

### Ratiometric fluorescence imaging of cysteine in living cells with **HDFL-Cys**

**Fig. 5:**
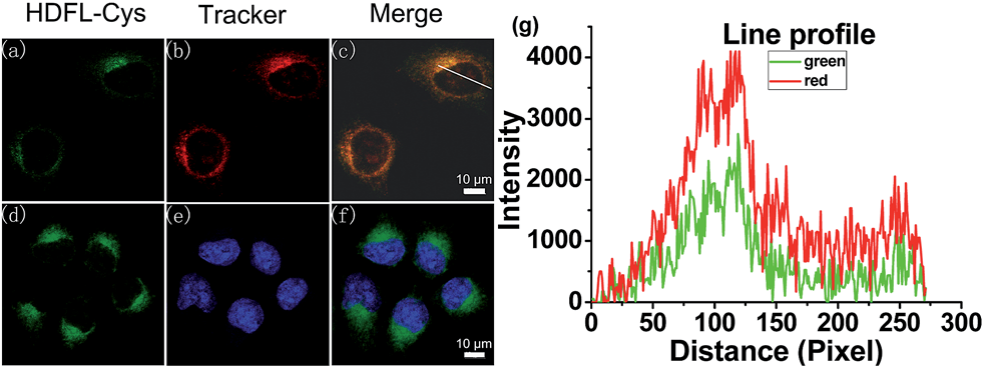
Confocal fluorescence images of HeLa cells co-stained with **HDFL-Cys** and a tracker. (a–c) Cells pre-treated with **HDFL-Cys** (5 μM) for 30 min and subsequently Mito-Tracker Red (1 μM) at 37 °C for 20 min. (d–f) Cells pre-treated with **HDFL-Cys** (5 μM) for 30 min at 37 °C and subsequently treated with Hoechst 33342 (1 μM) for 20 min; (a and d) images from **HDFL-Cys**, *λ*_ex_ = 488 nm and *λ*_em_ = 500–550 nm; (b) Fluorescence images from Mito-Tracker Red, *λ*_ex_ = 561 nm and *λ*_em_ = 570–620 nm; (e) Fluorescence images from Hoechst 33342, *λ*_ex_ = 405 nm and *λ*_em_ = 425–475 nm; (c) Overlay of the green and red channels; (f) Overlay of the blue and green channels. (g) Line profile: intensity profile of the white line in image overlap. Scale bars: 10 μm.

Encouraged by the above favourable spectral response of **HDFL-Cys** to cysteine *in vitro*, we further assessed the capability of the probe for monitoring cysteine in living cells. Prior to that, the cell cytotoxicity of **HDFL-Cys** was measured by standard MTT assays. The experimental results indicated that **HDFL-Cys** possesses low cytotoxicity to living cells (Fig. S22†). Meanwhile, colocalization experiments showed that **HDFL-Cys** penetrated the cell membrane well and selectively localized in mitochondria (Fig. 5 and S23†). In contrast, **HDFL1** distributed throughout the whole cells (Fig. S23 and 24†). This result was consistent with our hypothesis that **HDFL-Cys** was positively charged and localized in mitochondria, while **HDFL1** had no charge and targetable ability. Moreover, co-localization of **HDFL-Cys** and compound **R1** (a blue emission mitochondrial reagent) in HeLa cells further confirmed that **HDFL-Cys** indeed can be selectively localized in mitochondria (Fig. S25 and 26†).

**Fig. 6:**
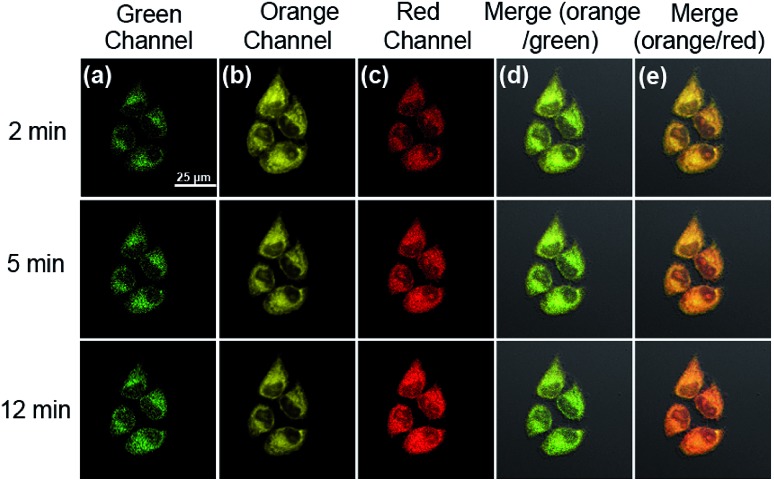
Fluorescence images of **HDFL-Cys** responding to Cys in living HepG2 cells along with reaction time by confocal fluorescence imaging. (a) and (c) are the images of **HDFL-Cys**; (b) is the image of Mito-Tracker Red; (d) and (e) are the merged images of (a) and (b), and (b) and (c), respectively. Fluorescence images were captured from the green channel of 500–550 nm (second column) and red channel of 663–738 nm (fourth column) with excitation at 488 nm and 640 nm. Third column: the Mito-Tracker Red (1 μM) was captured from the orange channel of 570–620 nm with excitation at 561 nm. Scale bar: 25 μm.

With this in mind, we next investigated whether **HDFL-Cys** can be used for the detection of cysteine in the mitochondria of living cells. Our goal is that **HDFL-Cys** first accumulates in mitochondria and then reacts with cysteine. As a result, we can see that the reaction product (dye **HDFL1**) accumulates only in mitochondria. As shown in Fig. S27,† with 30 min incubation, **HDFL-Cys** could detect both exogenous and endogenous cysteine in living cells with ratio imaging. The fluorescence intensity ratio values (*I*_red_/*I*_green_) were calculated by division of the intensity of the red channel to the green channel (Fig. S28†). However, the production of **HDFL-Cys** in response to cysteine was not only distributed in mitochondria. This may be ascribed to the overlong incubation time, so that **HDFL1** escaped from mitochondria. In view of this, we next carried out the real-time imaging of cysteine in living HepG2 cells for a short time (2–12 min). As shown in Fig. S29† and 6, after 2 min incubation, the fluorescence of Mito-Tracker Red (Orange channel, Fig. S29c† and 6b) was perfectly colocalized with the fluorescence of **HDFL-Cys** responding to cysteine (Red channel, Fig. S29f† and 6e). This phenomenon confirmed that **HDFL-Cys** indeed accumulated in mitochondria firstly and then reacted with cysteine, so that **HDFL1** localized in mitochondria. To the best of our knowledge, this is the first mitochondrial targetable probe to prove this. In addition, we can clearly see that the fluorescence of the red channel significantly enhanced with the extension of time (Fig. S29d† and 6c); however, the production of the probe, **HDFL1**, gradually escaped from mitochondria (Fig. 6e, S29d and f†). These findings are in good agreement with our above investigation. Simultaneously, a significant fluorescence increase in the ratio channel (*I*_red_/*I*_green_) was also observed over extended time (Fig. S29g and 30†). Additionally, the ratiometric probe **HDFL-Cys** exhibits accurate positioning in mitochondria throughout the whole journey (Fig. 6a and d). The above data confirm that **HDFL-Cys** can indeed target mitochondria and detect mitochondrial cysteine in living cells.

**Fig. 7:**
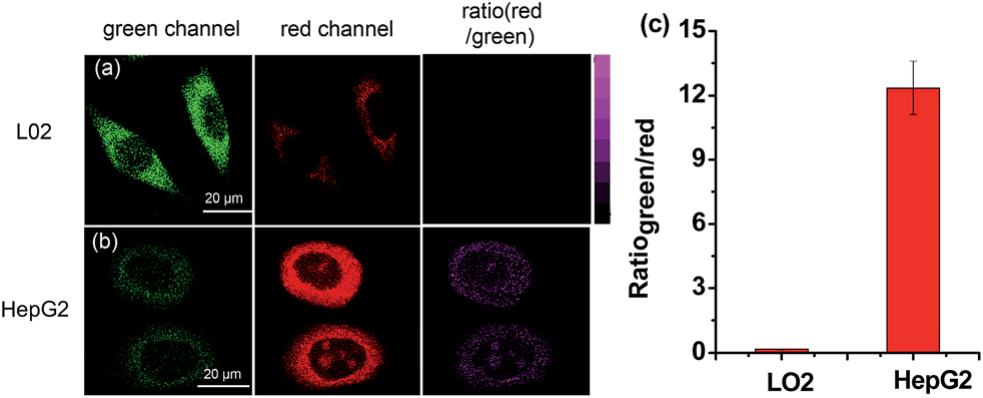
Fluorescence images of the probe responding to Cys in living HepG2 and L02 cells by confocal fluorescence imaging. Cells were incubated with 5 μM probe for 5 minutes. The fluorescence images were captured from the green channel of 500–550 nm (first column) and red channel of 663–738 nm (second column) with excitation at 488 nm and 640 nm. Third column: ratiometric images of the red channel to the green channel; fluorescence intensity ratios (*I*_red_/*I*_green_) in panels for L02 cells and HepG2 cells. Data are expressed as mean ± SD of three parallel experiments.

Inspired by the above results, we further examined whether **HDFL-Cys** could be used to distinguish between normal cells and cancer cells by imaging cysteine levels. It is well known that there is more reactive oxygen species (ROS) stress in cancer cells than normal cells. Therefore, cancer cells produce more reducing substances, such as GSH, Cys and Hcy, to maintain redox homeostasis.^46^,^47^ As shown in Fig. 7, L02 cells (normal liver cells) and HepG2 cells (cancer liver cells) were chosen for incubation with **HDFL-Cys**. It can be clearly seen that, with 5 min incubation, HepG2 cells showed very weak fluorescence in the green channel and strong fluorescence in the red channel (Fig. 7b). In contrast, L02 cells showed a brighter fluorescence in the green channel than that in the red channel (Fig. 7a). The ratio of two channels (*I*_red_/*I*_green_) showed an approximately 75-fold difference (Fig. 7c), indicating that HepG2 had a much higher level of cysteine than L02 cells. Therefore, **HDFL-Cys** was capable of distinguishing between the normal and cancer cells by monitoring the cysteine level.

## Conclusions

In summary, we have successfully developed innovative NIR dyes **HDFL** with excellent optical properties. The experimental results showed that **HDFL** dyes not only offer high chemical stability and photo-stability, together with biocompatibility for bioimaging, but also can be exploited as a novel platform for designing ratiometric fluorescent probes with large shifted emission bands through easy modifications on the hydroxyl group (>120 nm). Inspired by their unique properties, as a proof of concept, we designed a new type of mitochondrial probe **HDFL-Cys** for cysteine with simple one-step chemistry. This probe not only has a fast response to cysteine (400 s) with ratiometric emission, but also exhibits great sensitivity and selectivity. The imaging experiments demonstrated that **HDFL-Cys** can first accumulate in mitochondria and then react with the analyte (cysteine). This is the first mitochondrial probe to demonstrate this. Furthermore, **HDFL-Cys** can also be used to distinguish between normal cells and cancer cells by monitoring their cysteine levels. We expect that the new ICT dye **HDFL** will provide great potential for the development of ratiometric probes, especially mitochondrial ratiometric probes.

## Conflicts of interest

There are no conflicts to declare.

## Supplementary Material

Supplementary information
