# Supplementary material for: Detection of analytes in mitochondria without interference from other sites based on an innovative ratiometric fluorophore
Source: Chem Sci. 2018 May 22;9(24):5461–6. doi: 10.1039/c8sc01673a (PMC6011035; doi:10.1039/c8sc01673a)
Supplement: Supplementary file 1 [file SC-009-C8SC01673A-s001.pdf]

Electronic Supplementary Material (ESI) for Chemical Science.
This journal is © The Royal Society of Chemistry 2018

Supporting Information for

Detection of analytes in mitochondria without interference from other sites based on an innovative ratiometric fluorophore

Tian-Bing Ren,[ǂa] Qian-Ling Zhang,[ǂa] Dongdong Su,[b] Xing-Xing Zhang,[a] Lin Yuan,*[a] Xiao-Bing Zhang[a]

[a] State Key Laboratory of Chemo/Biosensing and Chemometrics, College of Chemistry and Chemical Engineering, Hunan University, Changsha 410082 (PR China).
E-mail: lyuan@hnu.edu.cn

[b] College of Chemistry and Chemical Engineering, Tianjin University of Technology, Tianjin 300384 (PR China).

ǂ These authors contributed equally to this work.

Table of contents:

1 **Materials and instruments…………………………………………...S3**

2 **Synthesis of compounds…………………………………………S4-5**

3 **Additional spectras……………………………………………S5-13**

4 **Supplemental tables…………………………………………..S13-14**

5 **Fluorescence Microscopic Studies…………………………...S15-18**

6 **NMR spectra and MS Spectra……………………………….S19-23**

1. Materials and instruments

Materials and instruments. Unless otherwise stated, all reagents were purchased from commercial suppliers and used without further purification. Solvents were purified by standard methods prior to use. Twice-distilled water was used throughout all experiments. Low resolution mass spectra were performed using an LCQ Advantage ion trap mass spectrometer from Thermo Finnigan or Agilent 1100 HPLC/MSD spectrometer. High-resolution electronspray (ESI-HRMS) mass spectra were recorded with a Bruker Q-TOF Qualification Standard Kit. NMR spectra were recorded on Bruker-400, using TMS as the internal standard. UV–Vis spectra were recorded on a UV-1800 spectrophotometer (Shimadzu Corporation, Japan). One-photon photoluminescence spectra were recorded on a HITACHI F4600 fluorescence spectrophotometer with a 1 cm standard quartz cell. Cell imaging was performed on Nikon A1plus confocal microscope. The pH measurements were carried out on a PHS-3C pH meter (INESA instrument). TLC analysis was performed on silica gel plates and column chromatography was conducted over silica gel (mesh 200–300) columns, obtained from the Yantai Jiangyou silica gel Development Company Limited.

Calculation of fluorescence quantum yield. Fluorescence quantum yield was determined using optically matching solutions of cresol purple (Φ_f = 0.54 in methanol), ICG (Φ_f = 0.13 in DMSO)[1] or rhodamine 6G (Φ_f = 0.95 in EtOH)[2] as the standard and the quantum yield was calculated using the following equation:

$$\Phi_s = \Phi_r(A_r F_s / A_s F_r)\ (n_s^2/n_r^2)^2$$

where, s and r denote sample and reference, respectively. A is the absorbance. F is the relative integrated fluorescence intensity and n is the refractive index of the solvent.

Calculation of p*Ka* Values. pKa values of **HDFL1** and **HDFL2** dye at acidic to near-neutral pH regions were calculated by regression analysis of the fluorescence data to fit equation:

$$pH - pKa = \log (F_{max} - F)/(F - F_{min})$$

Where F is the area under the corrected emission curve, F_{max} and F_{min} are maximum and minimum limiting values of F, respectively.

Detection limit calculation. The detection limit was calculated based on the fluorescence titration. The detection limit is calculated using the following equation:

$$3\sigma/k$$

where σ is the standard deviation of blank measurements, k is the slope between the emission ratios versus sample concentration.

2. Synthesis of compounds

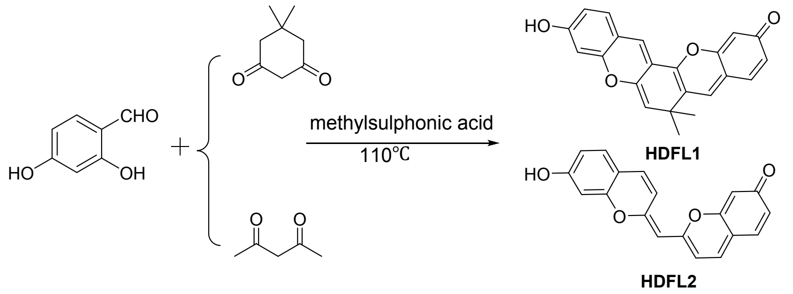

Scheme S1. The systhesis of **HDFL1** and **HDFL2**.

HDFL1. Beta-resorcylaldehyde (2.0 mmol, 276.0 mg) and dimedone (1.0 mmol, 140.1 mg) were dissolved in methylsulphonic acid (3.0 mL), then the reaction mixture was heated to 110 °C and keep at this temperature for 2 hour. The obtained reaction solution was cooled to room temperature, and then poured onto crushed ice (100.0 g). Perchloric acid (70%, 2.0 mL) was then added, and the resulting precipitate was filtered off , washed with cold water (50.0 mL), and then the crude product was purified via chromatography (silica gel, CH_2Cl_2/CH_3CH_2OH = 25:1) to get a red solid product. Yield 62.0 mg (18.0%). ^{1}H NMR (400 MHz, MeOD) δ 8.21 (s, 2H), 7.71 (s, 1H), 7.69 (s, 1H), 7.04 (d, J = 2.2 Hz, 1H), 7.02 (d, J = 2.2 Hz, 1H), 6.98 (2H), 6.47 (s, 1H), 1.72 (s, 6H). ^{13}C NMR (100 MHz, DMSO) δ 167.4, 165.3, 154.5, 138.0, 130.9, 130.1, 117.9, 115.7, 102.4, 96.3, 56.5, 33.1. HRMS (ESI): for $C_{22}H_{17}O_4$: m/z = 345.1225 $[M + H]^+$.

HDFL2. Beta-Resorcylaldehyde (2.0 mmol, 276.0 mg) and diacetone (1.0 mmol, 100.2 mg) were dissolved in methylsulphonic acid (3.0 mL). The reaction mixture was heated to 110 °C and keep at this temperature for 2 hour. The obtained reaction solution was cooled to room temperature, and then poured onto crushed ice (100.0 g). Perchloric acid (70%, 2.0 mL) was then added, and the resulting precipitate was filtered off and washed with cold water (50.0 mL), and then the crude product was purified via chromatography (silica gel, CH_2Cl_2/CH_3CH_2OH = 25:1) to get solid product. Yield 55.2 mg (18.2%). ^{1}H NMR (400 MHz, MeOD) δ 8.02 (2H), 7.80 (2H), 7.61 (2H), 7.03 (2H), 7.00 (1H), 6.98 (1H), 6.11 (1H). ^{13}C NMR (100 MHz, MeOD) δ 166.2, 156.4, 141.9, 130.3, 118.1, 114.8, 102.3, 95.6. HRMS (ESI): for $C_{19}H_{13}O_4$: m/z = 305.0889 $[M + H]^+$.

HDFL-Cys. **HDFL1** (1.0 mmol, 345.0 mg) and acryloyl chloride (1.5 mmol, 122.0 μL) were dissolved in anhydrous acetonitrile (5.0 mL), and the reaction mixture were stirred at room temperature for 5 hour, and then all solvent was evaporated. The crude product was purified via chromatography (silica gel, CH_2Cl_2) to get **HDFL-Cys** (228.2 mg) with a yield of about 50.4%. ^{1}H NMR (400 MHz, $CDCl_3$) δ 8.59 (2H), 8.15 (2H), 7.53 (2H), 7.35 (2H), 6.69 (2H), 6.52 (1H), 6.35 (2H), 6.14 (2H), 1.87 (6H). ^{13}C NMR (100 MHz, $CDCl_3$) δ 170.0, 169.8, 163.3, 154.9, 152.6, 139.9, 134.5, 134.2, 131.4, 127.1, 121.8, 120.4, 110.0, 33.1, 29.7. MS (ESI): for $[C_{28}H_{21}O_6]^+$: m/z = 453.1 $[M]^+$.

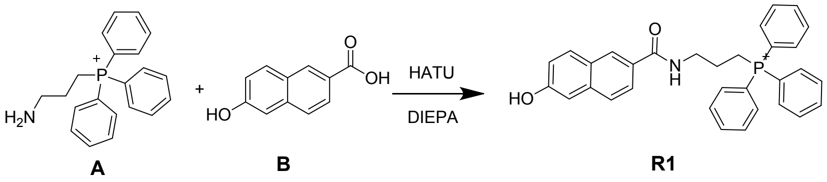

Compound R1. A mixture of compound A (320 mg, 1 mmol), compound B (188 mg, 1 mmol), HATU (420 mg, 1.1 mmol) and catalytic amount of DIEPA was dissolved in DMF (5 mL) and stirred for 4 h at room temperature. After completion of reaction, the solution was poured into water. The resulting precipitate was filtered off and washed with plenty of water. After the sample dried, purification by flash column chromatography to obtain compound R1 as a white powder. We characterized its structure by mass spectral analysis. MS (ESI): calcd for $[C_{32}H_{29}NO_2P]^+$ 490.2, found 490.2.

3. Additional spectras

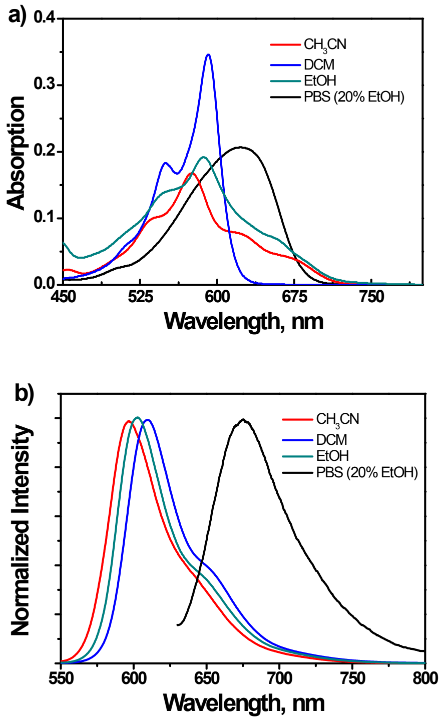

Figure S1. The absorption (a) and normalized fluorescence emission spectra (b) of **HDFL1** (5 µM) in different solvent.

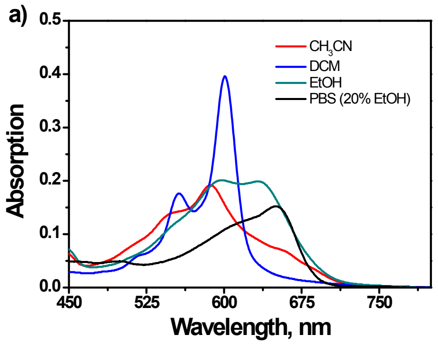

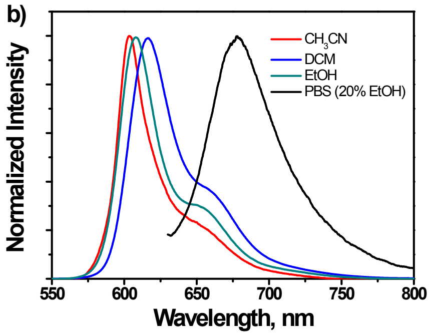

Figure S2. The absorption (a) and normalized fluorescence emission spectra (b) of **HDFL2** (5 μM) in different solvent.

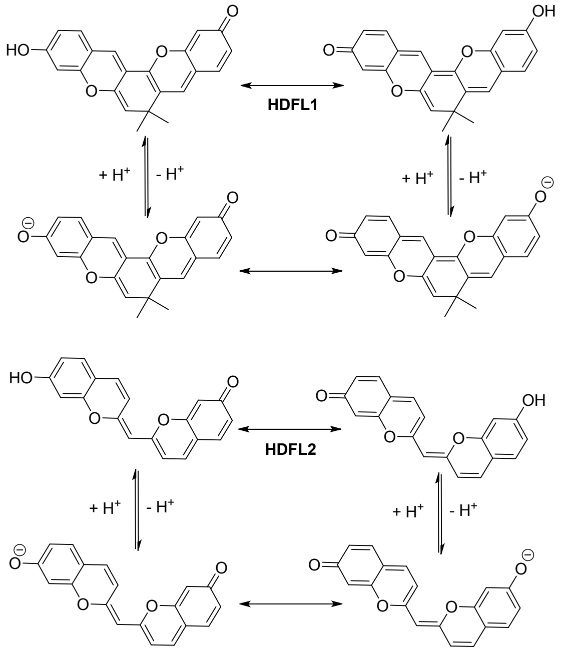

Scheme S2. The dissociation and vibrational states of **HDFL1-2** in polar solutions.

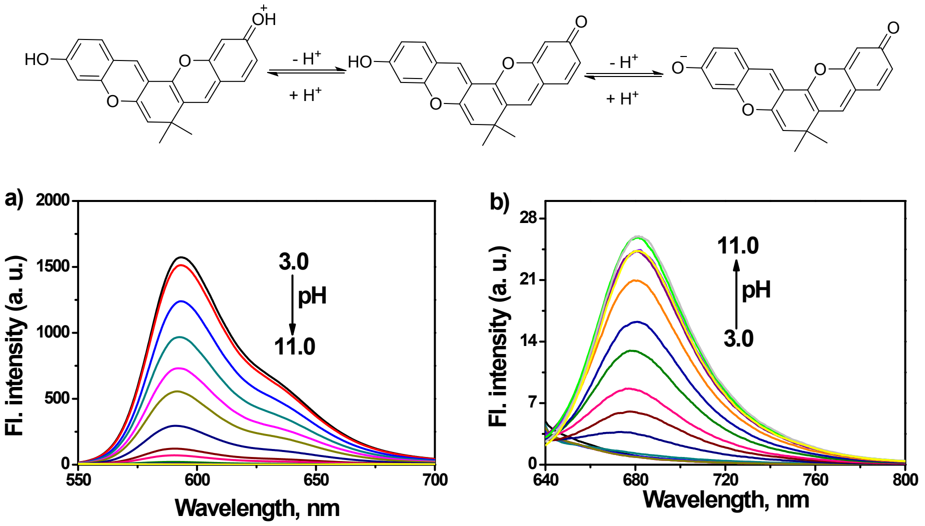

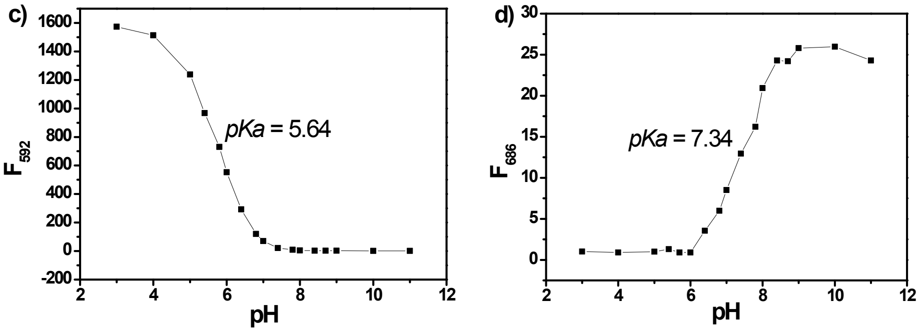

Figure S3. Fluorescence emission spectra of **HDFL1** (5 μM) in PBS buffer (containing 20% EtOH) solution with pH changing from 3.0 to 11.0, (a) λ_{ex}: 520 nm; (b) λ_{ex}: 620 nm. Sigmoidal fitting of the pH-dependent fluorescence intensity at 592 nm (c) and 681 nm (d) of **HDFL1.**

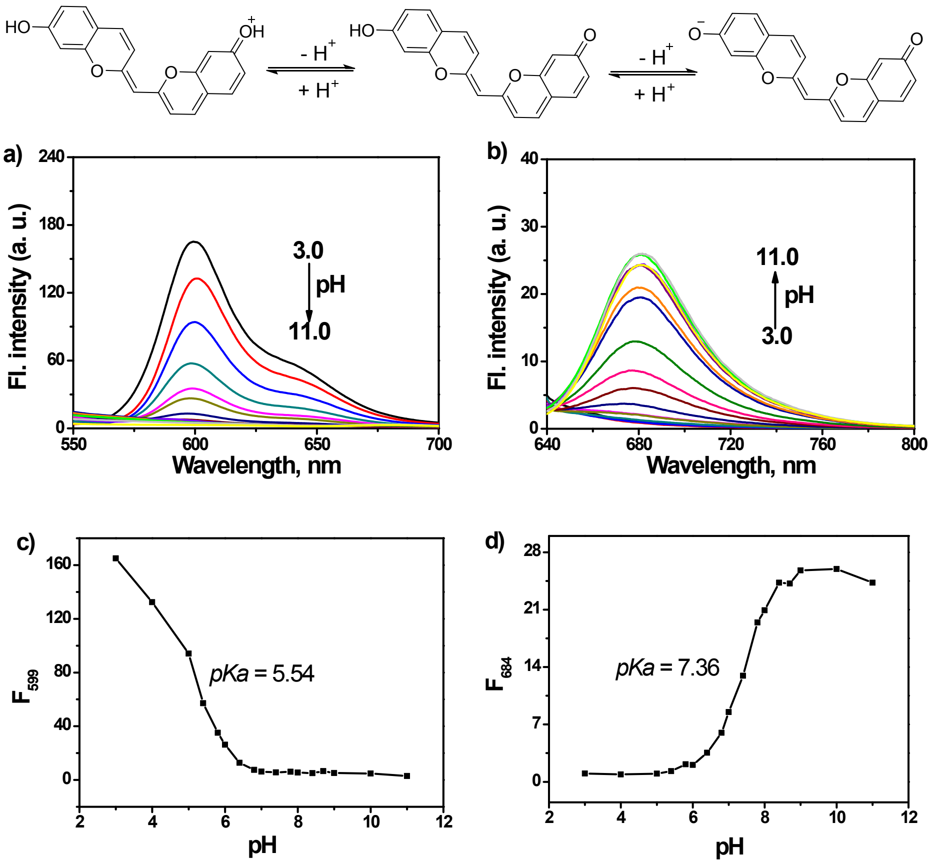

Figure S4. Fluorescence emission spectra of **HDFL2** (5 μM) in PBS buffer (containing 20% EtOH) solution with pH changing from 3.0 to 11.0, (a) λ_{ex}: 520 nm; (b) λ_{ex}: 620 nm. Sigmoidal fitting of the pH-dependent fluorescence intensity at 599 nm (c) and 684 nm (d) of **HDFL2**.

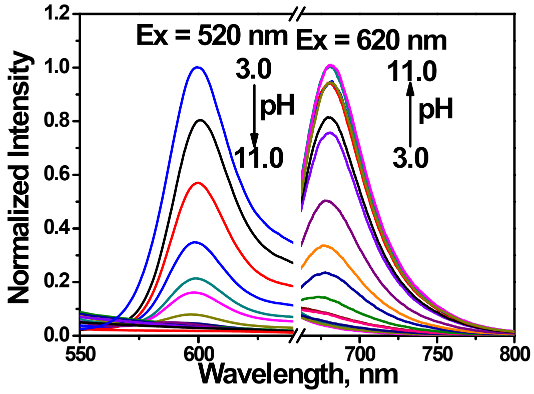

Figure S5. The normalized emissiom intensity of **HDFL2** (5 μM) in PBS buffer (containing 20% EtOH) solution with pH changing from 3.0 to 11.0.

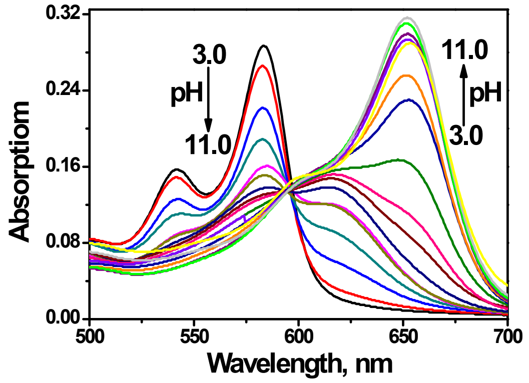

Figure S6. The absorption spectra of **HDFL2** (5 μM) in PBS buffer (containing 20% EtOH) solution with pH changing from 3.0 to 11.0.

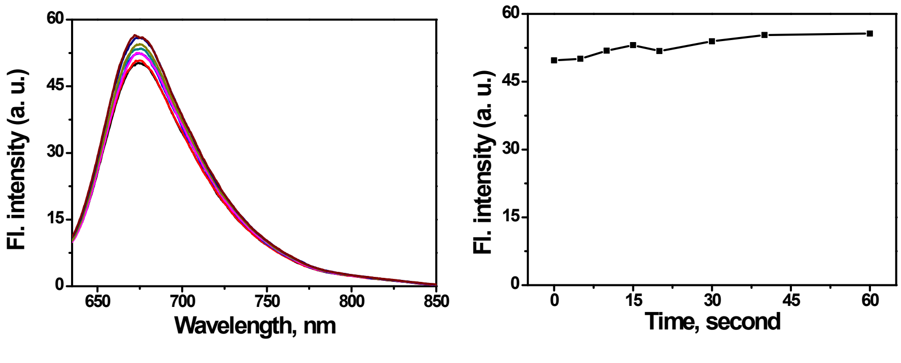

Figure S7. Time-dependent fluorescence emission spectra of **HDFL1** (5 μM) in 7.4 PBS (containing 20% EtOH and 1 mM GSH) for 0-60 min.

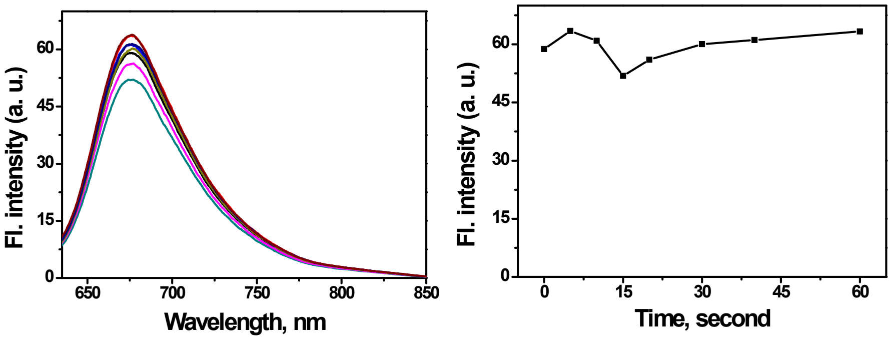

Figure S8. Time-dependent fluorescence emission spectra of **HDFL1** (5 μM) in 7.4 PBS (containing 20% EtOH and 0.1 mM H_2O_2) for 0-60 min.

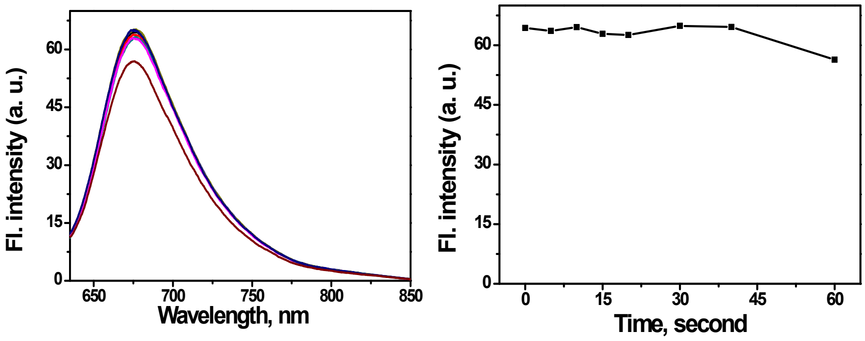

Figure S9. Time-dependent fluorescence emission spectra of **HDFL1** (5 μM) in 7.4 PBS (containing 20% EtOH and 0.1 mM H_2S) for 0-60 min.

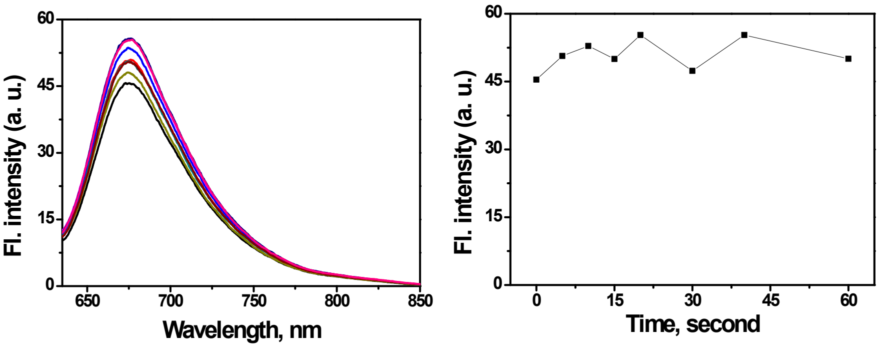

Figure S10. Time-dependent fluorescence emission spectra of **HDFL1** (5 μM) in 7.4 PBS (containing 20% EtOH and 0.1 mM HOCl) for 0-60 min.

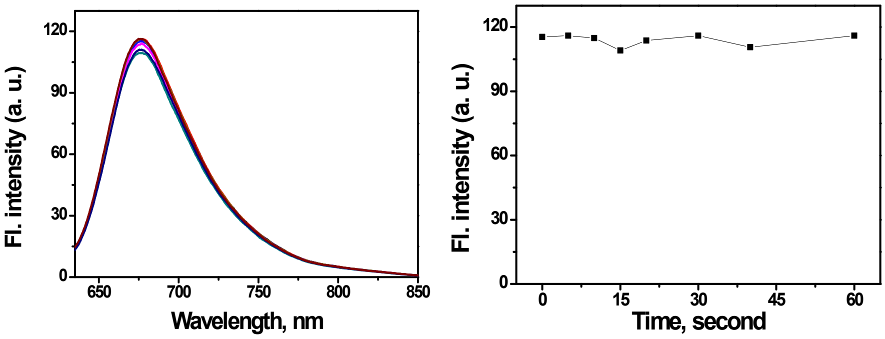

Figure S11. Time-dependent fluorescence emission spectra of **HDFL1** (5 μM) in 7.4 PBS (containing 20% EtOH and 25 μM $ONOO^{-}$) for 0-60 min.

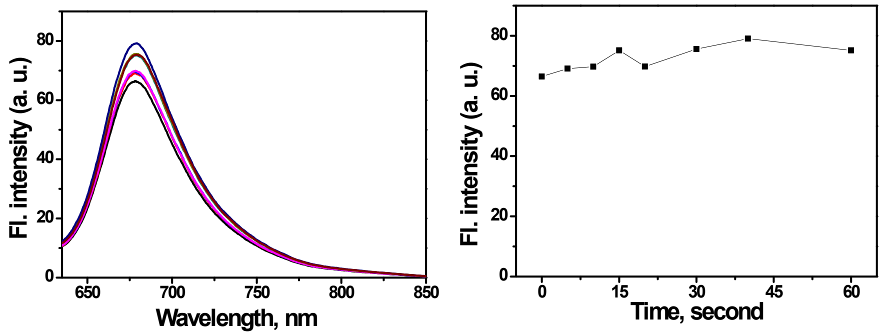

Figure S12. Time-dependent fluorescence emission spectra of **HDFL2** (5 μM) in 7.4 PBS (containing 20% EtOH and 1 mM GSH) for 0-60 min.

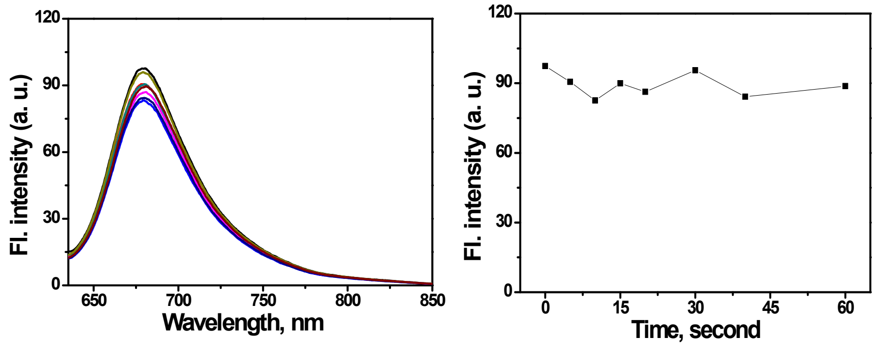

Figure S13. Time-dependent fluorescence emission spectra of **HDFL2** (5 μM) in 7.4 PBS (containing 20% EtOH and 0.1 mM H_2O_2) for 0-60 min.

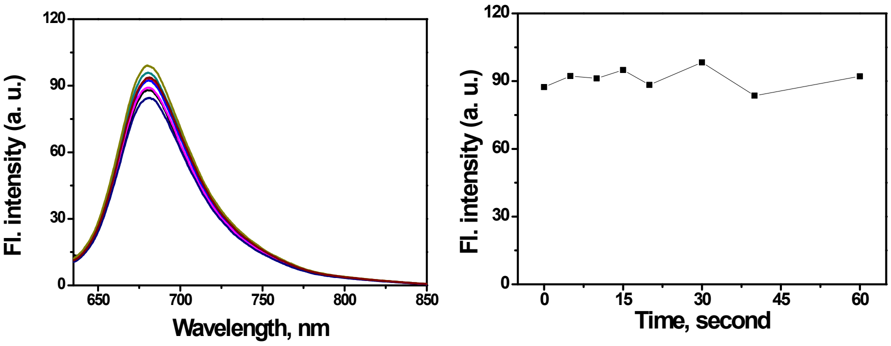

Figure S14. Time-dependent fluorescence emission spectra of **HDFL2** (5 μM) in 7.4 PBS (containing 20% EtOH and 0.1 mM H_2S) for 0-60 min.

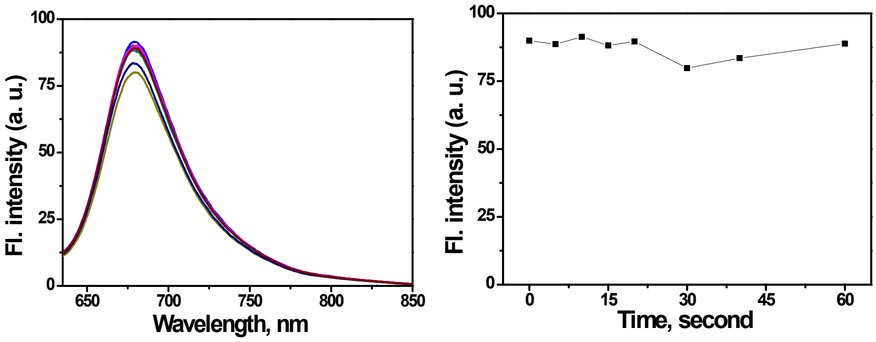

Figure S15. Time-dependent fluorescence emission spectra of **HDFL2** (5 μM) in 7.4 PBS (containing 20% EtOH and 0.1 mM HOCl) for 0-60 min.

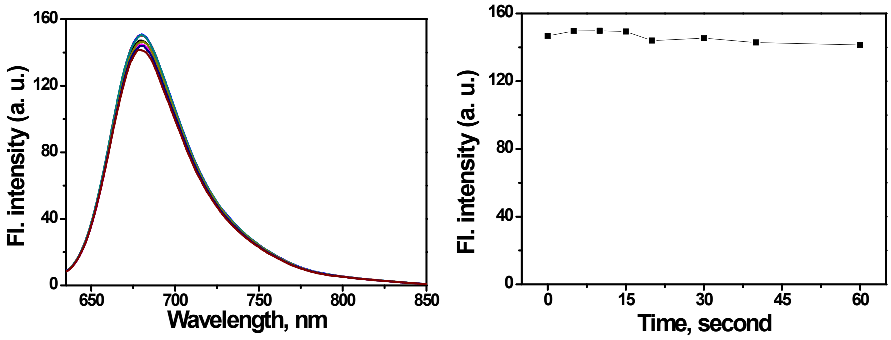

Figure S16. Time-dependent fluorescence emission spectra of **HDFL2** (5 μM) in 7.4 PBS (containing 20% EtOH and 25 μM $ONOO^-$) for 0-60 min.

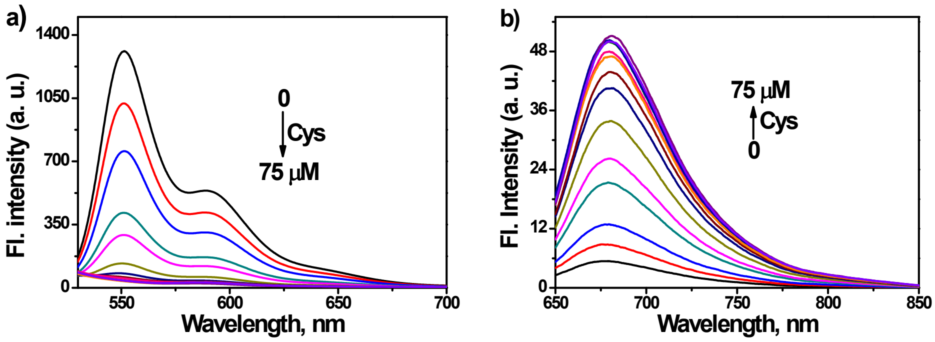

Figure S17. Fluorescence emission spectra fluorescent probe **HDFL-Cys** (5 μM) in the presence of various concentrations of Cys (0−75 μM) in PBS (25 mM, pH = 7.4, 20% EtOH), (a) λ_{ex}: 520 nm; (b) λ_{ex}: 620 nm.

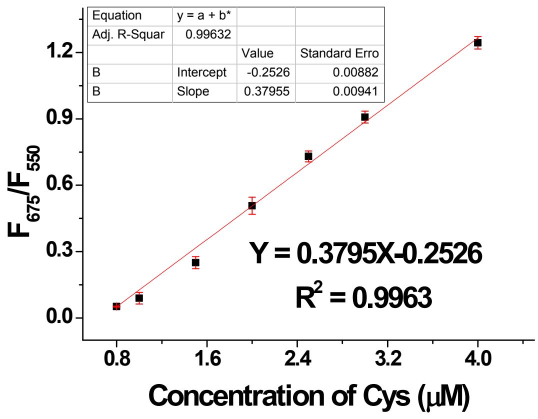

Figure S18. The linear correlation between fluorescence ratio change of **HDFL-Cys** (5 μM) and the concentrations of Cys.

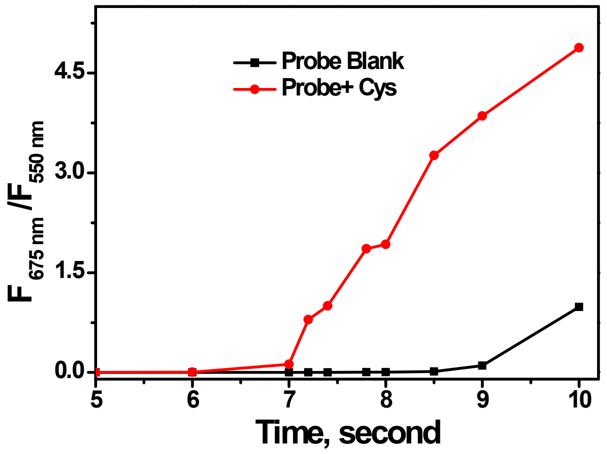

Figure S19. pH-dependent fluorescence intensity ratio changes of HDFL-Cys (5 μM) toward Cys (100 μM), in PBS buffer (25 mM, containing 20% EtOH, pH = 3-10), λ_{ex}: 520 nm and λ_{ex}: 620 nm.

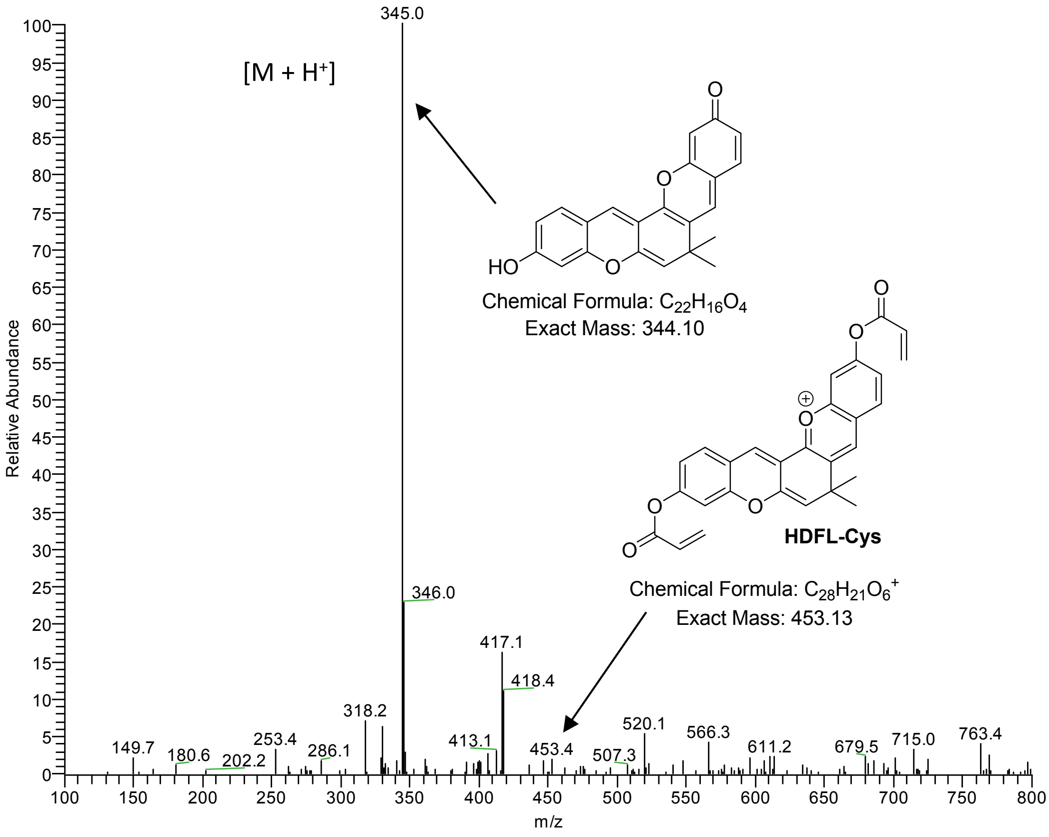

Figure. S20. ESI-MS spectrum of compound HDFL-Cys in the presence of Cys in pH 7.4 PBS buffer (containing 20% EtOH).

4. Supplemental tables

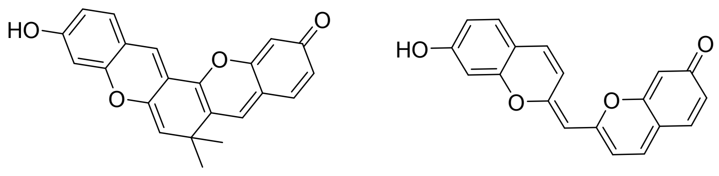

Table S1. Photo-physical Properties of **HDFL1**

	**DCM**	**CH_3CN**	**EtOH**	**PBS (containing 20% EtOH)**
$\lambda_{abs}/\lambda_{em}$	592nm/ 608 nm	574 nm/ 596 nm	585 nm/ 602 nm	624nm/ 681 nm
ε_{max}/$M^{-1}cm^{-1}$	6.89×10^4	3.35×10^4	3.85×10^4	4.16×10^4

Table S2. Photo-physical Properties of **HDFL2**.

	**DCM**	**CH_3CN**	**EtOH**	**PBS (containing 20% EtOH)**
$\lambda_{abs}/\lambda_{em}$	600 nm/ 616nm	584nm/ 603 nm	597 nm/ 608 nm	649nm/ 684 nm
ε_{max}/M^{-1}cm^{-1}	7.97×10^4	3.87×10^4	4.02×10^4	3.08×10^4

Table S3. Photo-physical Properties of **HDFL1-2**.[a]

**Comp.**	***Φ*(100%) (DCM)**	***Φ*(100%) (CH_3CN)**	***Φ*(100%) (EtOH)**	***Φ*(100%) (PBS, 20% EtOH)**
**HDFL1**	85.97	31.20	32.60	4.33
**HDFL2**	69.68	1.46	4.40	3.47

[a] All the measurements were carried out at 25°C. The relative fluorescence quantum yields in DCM, CH_3CN and EtOH were estimated using cresol purple (Φ_f = 0.53 in CH_3OH) and in PBS (20% EtOH) were estimated using ICG (Φ_f = 0.13 in DMSO)[1], as a fluorescence standard.

Table S4. Photo-physical Properties of **HDFL-Cys** in PBS (containing 20% EtOH).

	λ_{abs}	λ_{em}	ε_{max}/M^{-1}cm^{-1}	***Φ*(100%)**
**HDFL-Cys**	539 nm	551 nm	1.55×10^4	45.78

[a] All the measurements were carried out at 25°C. The relative fluorescence quantum yield in PBS (20% EtOH) were estimated using rhodamine 6G (Φ_f = 0.95 in EtOH)[2], as a fluorescence standard.

Table S5. Comparison of the response time of Cys probes

**Comp.**	**Solution**	**Response time**	**Reference**
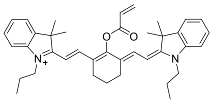	HEPES, 10% EtOH	1800 s	*Chem. Sci.*, 2012, **3**, 2760–2765
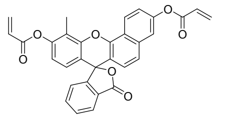	1 mM CTAB media HEPES buffer	900 s	*Org. Biomol. Chem.*, 2012, **10**, 2739–2741
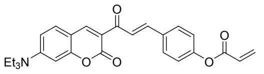	HEPES, 50% DMSO	1440 s	*Org. Lett.*, 2017, **19**, 82−85
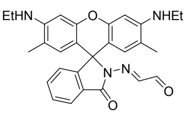	PBS, 30% EtOH	3000 s	*Chem. Commun.*, 2009, **39**, 5904–5906
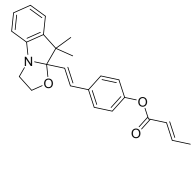	HEPES buffer	5400 s	*Anal. Chem.*, 2016, **88**, 7178−7182
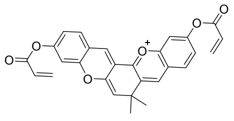	PBS, 20% EtOH	400 s	*This work*

5. Fluorescence Microscopic Studies

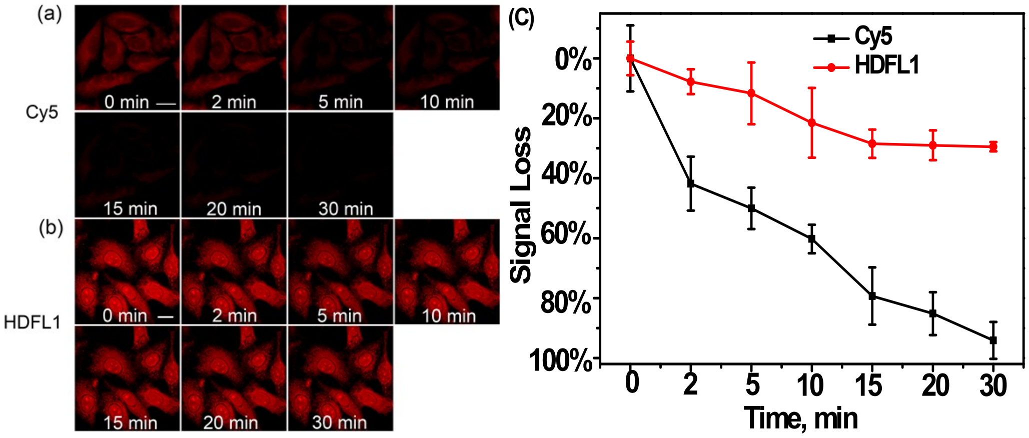

Figure S21. Confocal fluorescence images of HepG2 cells co-stained with Cy5 (5 μM) (a) or HDFL1 (5 μM) (b) with continuous irradiation for 30 min using confocal microscope with the same parameters. (c) Quantification of the relative mean fluorescence levels of cells from the images of (a) and (b). Scale bar = 20 μm. For Cy5 and HDFL-Cys, λ_{ex} = 640 nm, λ_{em} = 663-738 nm.

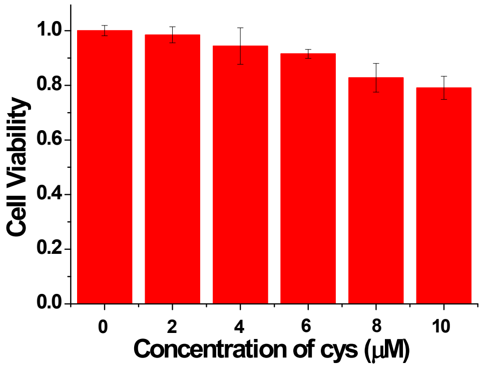

Figure S22. Cytotoxicity of **HDFL-Cys** (5 μM) for HeLa cells. Cells were incubated with **HDFL-Cys** at corresponding concentrations for 12 h. Cell viability was measured by MTT assay and the results are reported as percentage relative to untreated cells (mean ± SD).

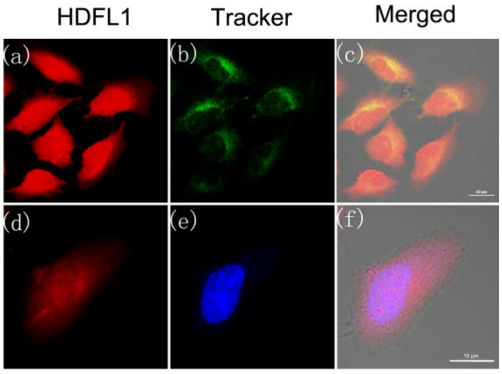

Figure S23. Confocal fluorescence images of HeLa cells co-stained with **HDFL1** and tracker. (a-c) Cells pre-treated with **HDFL1** (5 μM) for 30 min at 37 °C and then treated with Mito-tracker red (1 μM) for 20 min; (d-f) Cells pre-treated with **HDFL1** (5 μM) at 37 °C for 30 min and then treated with Hoechst 33342 (1 μM) for 20 min. (a, d) images from **HDFL1**, λ_{ex} = 640 nm, λ_{em} = 663-738 nm; (b)

Fluorescence images from Mito-tracker Red, λ_{ex} = 561 nm, λ_{em} = 570-620 nm; (e) Fluorescence images from Hoechst 33342, λ_{ex} = 405 nm, λ_{em} = 425-475 nm; (c) Overlay of the green and red channels. (f) Overlay of the blue and red channels. Scar bars= 10 μm.

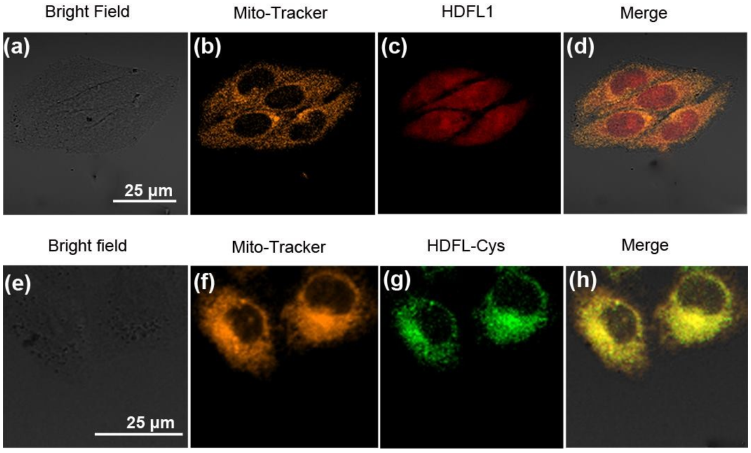

Figure S24. Confocal fluorescence images of HepG2 cells co-stained with **HDFL-Cys** or **HDFL1**. (a-d) Cells pre-treated with **HDFL1** (5 μM) for 30 min at 37 °C and then treated with Mito-tracker Red (1 μM) at 37 °C for 20 min; (e-h) Cells pre-treated with **HDFL-Cys** (5 μM) for 30 min and then treated with Mito-tracker Red (1 μM) at 37 °C for 20 min. (a, e) Bright field; (b, f) Fluorescence images from Mito-tracker Red, λ_{ex} = 561 nm, λ_{em} = 570-620 nm; (c) images from **HDFL1**, λ_{ex} = 640 nm, λ_{em} = 663-738 nm; (g) Fluorescence images from **HDFL-Cys**, λ_{ex} = 488 nm, λ_{em} = 500-550 nm; (d) Overlay of the orange and red channels; (f) Overlay of the orange and green channels. Scar bars = 25 μm.

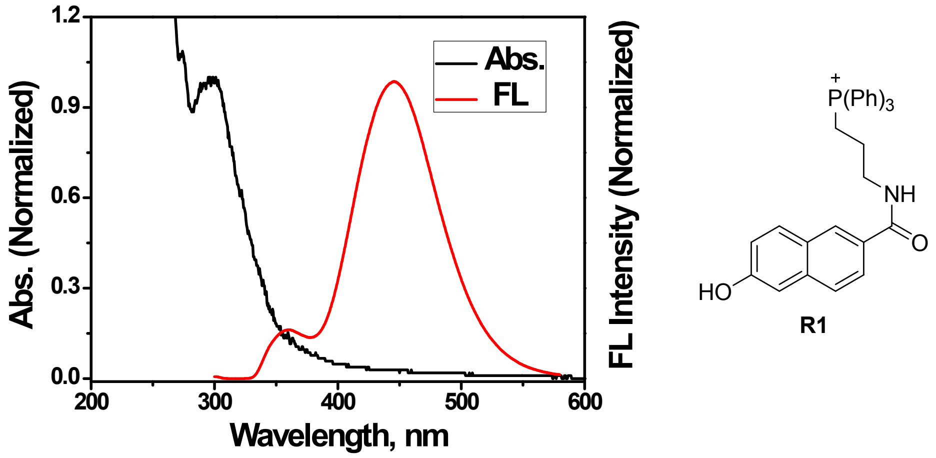

Figure S25. The normalized absorption and normalized fluorescence emission spectra of R1 (5 μM) in PBS buffer (pH = 7.4, containing 20% EtOH).

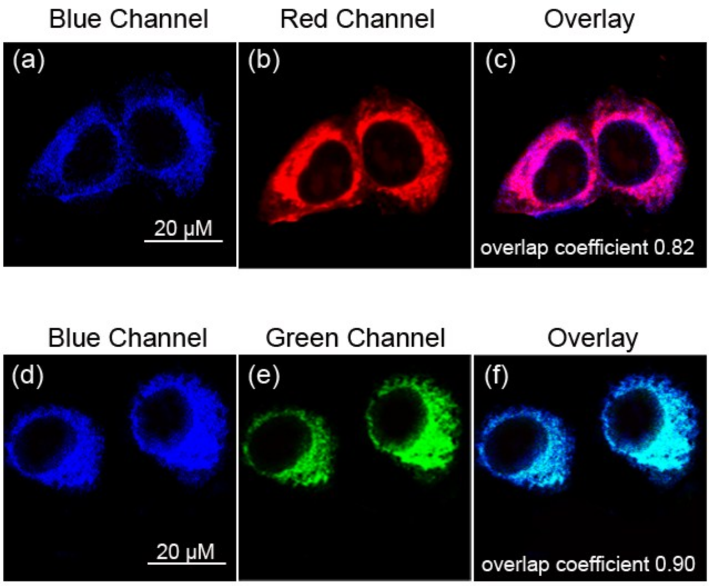

Figure S26. Confocal fluorescence images of Hela cells co-stained with HDFL-Cys or R1. (a-c) Cells pre-treated with R1 (5 μM) for 10 min at 37 °C and then treated with Mito-tracker Red (1 μM) at 37 °C for 10 min; (d-f) Cells pre-treated with R1 (5 μM) for 10 min and then treated with HDFL-Cys (5 μM) at 37 °C for 10 min. (a, d) Fluorescence images from R1, λ_{ex} = 405 nm, λ_{em} = 425-475 nm; (b images from Fluorescence images from Mito-tracker Red, λ_{ex} = 561 nm, λ_{em} = 570-620 nm;; (e) Fluorescence images from HDFL-Cys, λ_{ex} = 488 nm, λ_{em} = 500-550 nm; (c) Overlay of the blue and red channels; (f) Overlay of the blue and green channels. Scar bars = 20 μm.

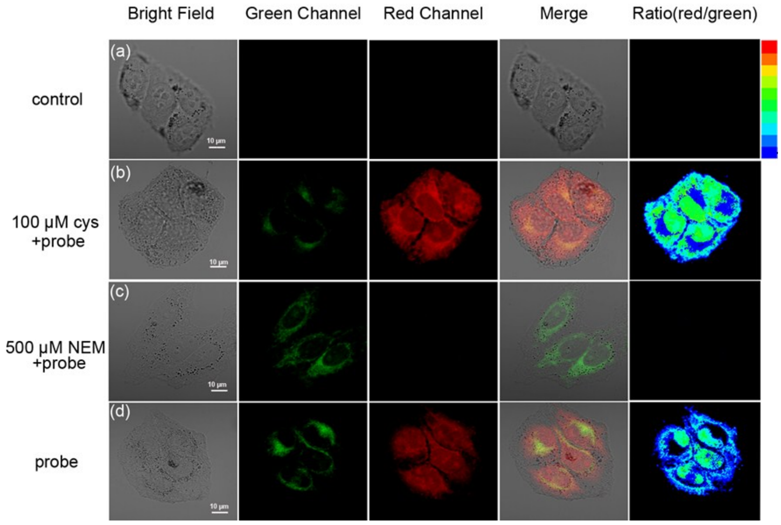

Figure S27. Fluorescence images of **HDFL-Cys** responding to Cys in living HepG2 cells by confocal fluorescence imaging. (a) Control experiment without **HDFL-Cys**; (b) Cells only treated with **HDFL-Cys** (5 μM) for 30 min at 37 °C. (c) Cells were pre-treated with NEM (500 μM) for 30 min, and then incubated with **HDFL-Cys** (5 μM) for another 30 min, (d) Cells were pre-treated with Cys (100 μM) for 30 min, and then incubated with **HDFL-Cys** (5 μM) for 30 min. The fluorescence images were captured from the green channel of 500-550 nm (second column) and red channel of 663-738 nm (third column) with excitation at 488 nm and 640 nm. Fifth column: ratiometric images of the red channel to the green channel. Scale bar: 10 μm.

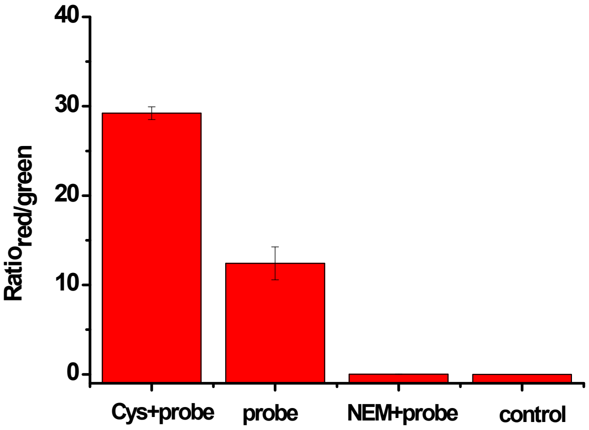

Figure S28. Fluorescence intensity ratios (I_{red}/I_{green}) in panels. Data are expressed as mean ± SD of three parallel experiments.

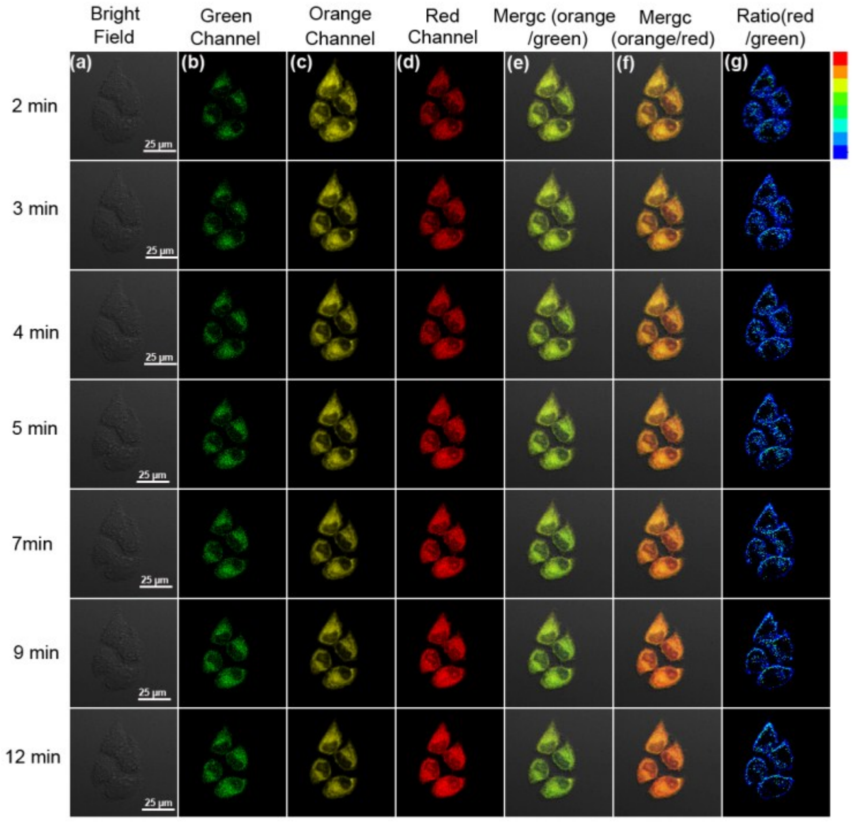

Figure S29. Fluorescence images of HDFL-Cys responding to Cys in living HepG2 cells along with reaction time by confocal fluorescence imaging. (a) Brightfield image of cells pretreated with Mito-Tracker Red (1 μM) for 20 min and followed by the addition of HDFL-Cys (5 μM); (b) and (d) are the image of HDFL-Cys; (c) is the image of Mito-Tracker Red; (e) and (f) are the merged image of (b) and (c), (c) and (d), respectively. Fluorescence images were captured from the green channel of 500-550 nm (second column) and red channel of 663-738 nm (fourth column) with excitation at 488 nm and 640 nm. Third column: the Mito-Tracker Red (1 μM) was captured from the orange channel of 570-620 nm with excitation at 561 nm. Scale bar: 25 μm.

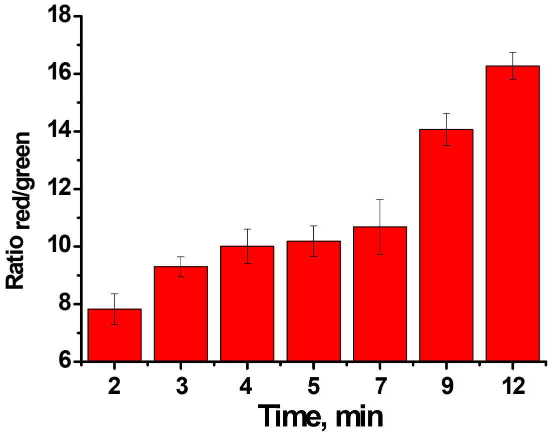

Figure S30. Fluorescence intensity ratios (I_{red}/I_{green}) in panels with reaction time. Data are expressed as mean ± SD of three parallel experiments.

6. MS Spectra and NMR spectra

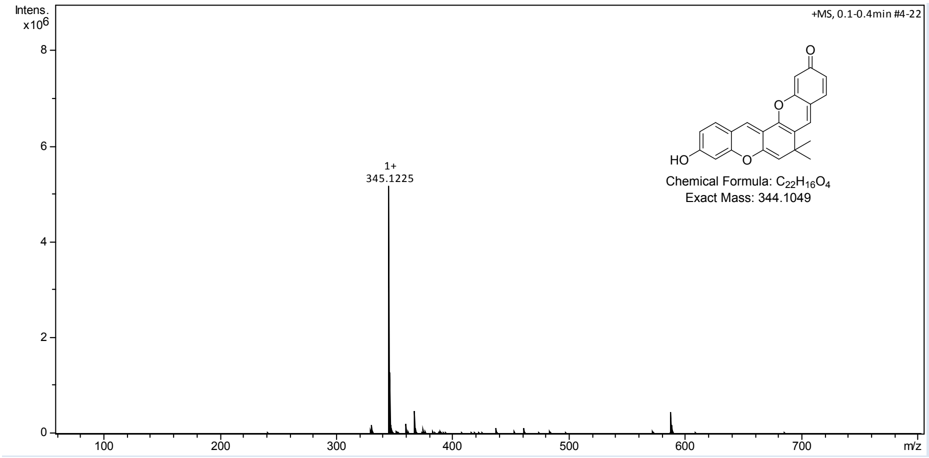

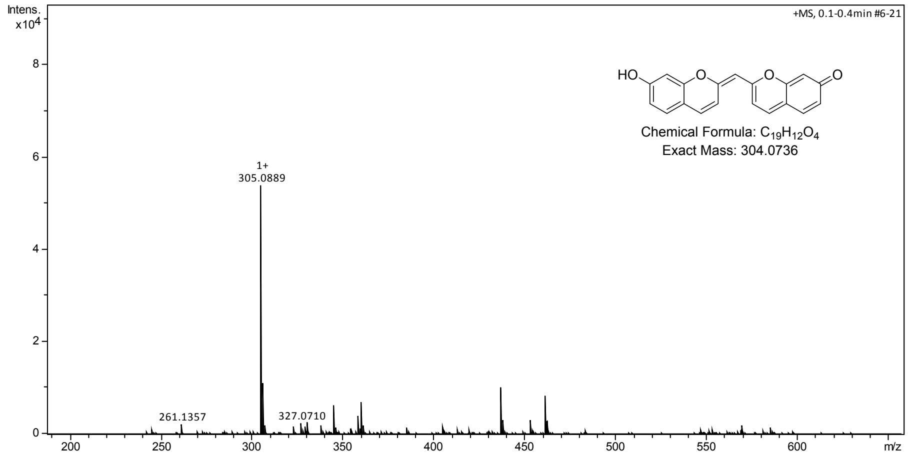

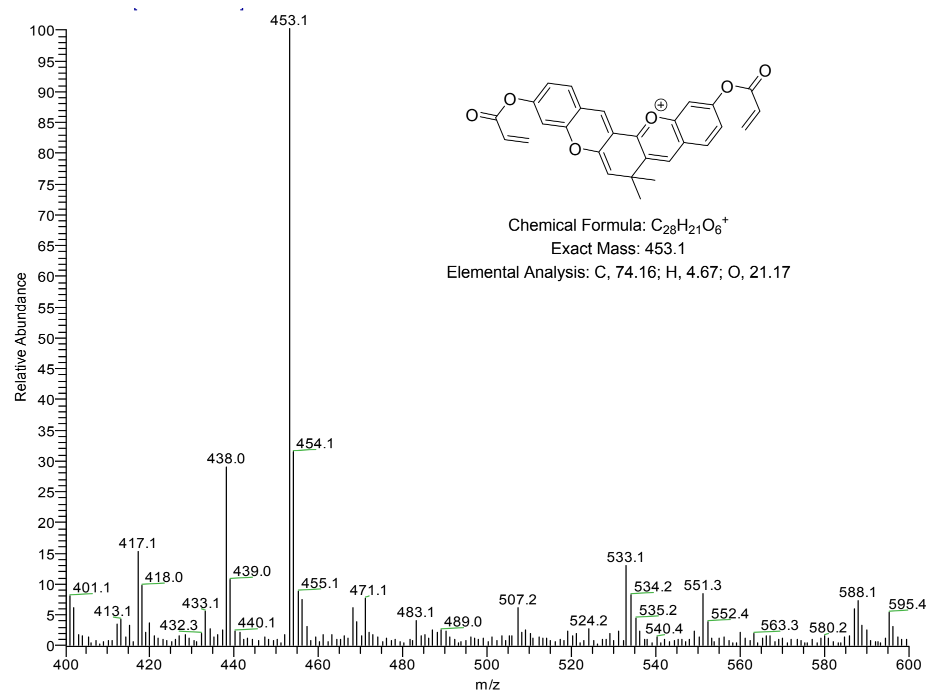
Chemical Formula: $C_{28}H_{21}O_6^+$
Exact Mass: 453.1
Elemental Analysis: C, 74.16; H, 4.67; O, 21.17
Relative Abundance
m/z
453.1
454.1
438.0
417.1
439.0
418.0
533.1
534.2
551.3
588.1
595.4
507.2

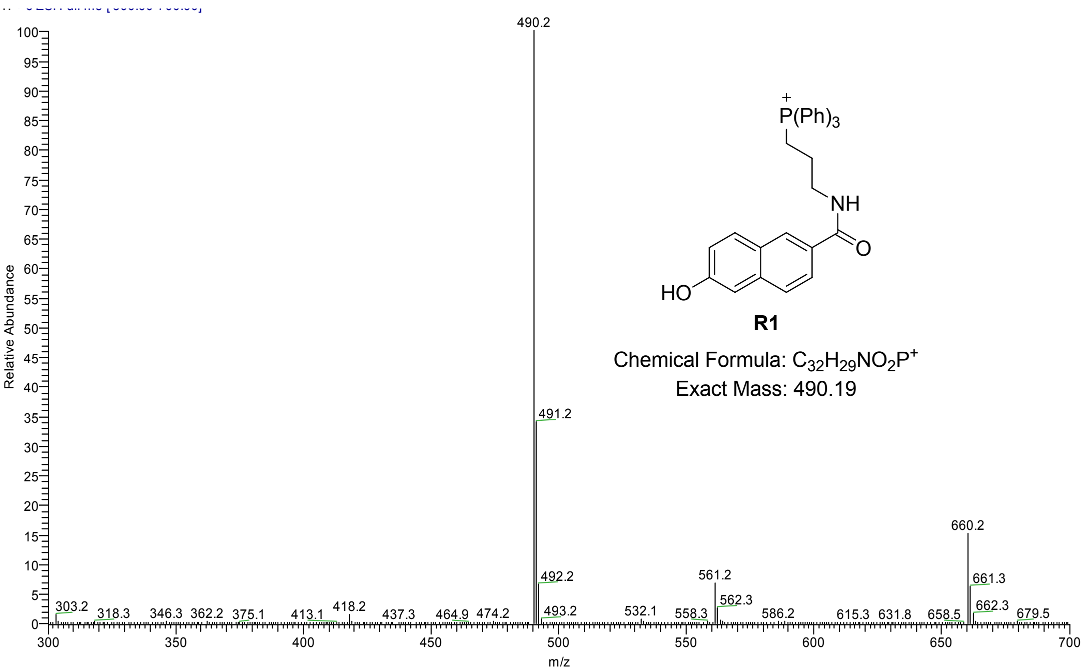
P(Ph)3
NH
O
HO
R1
Chemical Formula: $C_{32}H_{29}NO_2P^+$
Exact Mass: 490.19
Relative Abundance
m/z
490.2
491.2
492.2
660.2
661.3
561.2
562.3

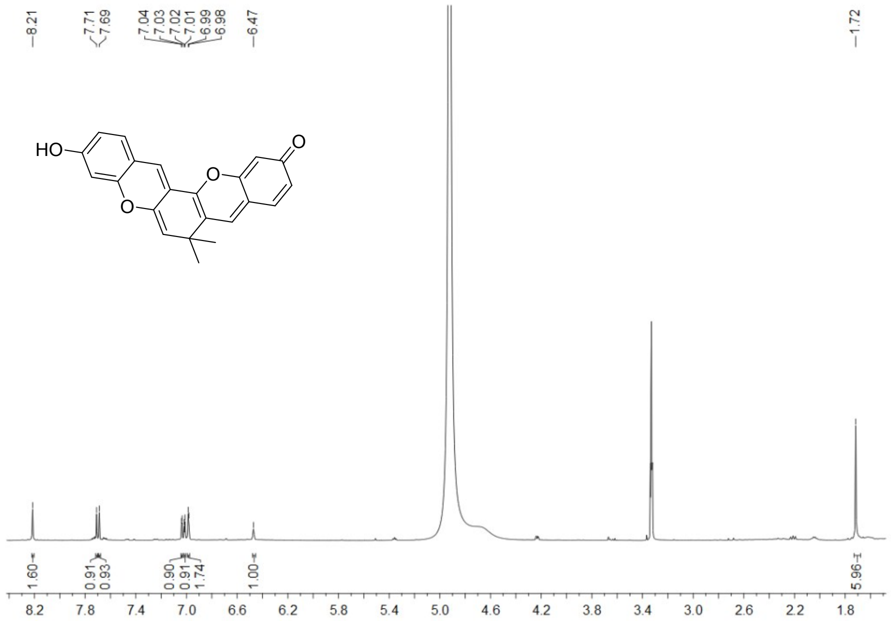

8.21
7.71
7.69
7.04
7.03
7.02
7.01
6.99
6.98
6.47
1.72
HO
O
1.60
0.91
0.93
0.90
0.91
1.74
1.00
5.96
8.2 7.8 7.4 7.0 6.6 6.2 5.8 5.4 5.0 4.6 4.2 3.8 3.4 3.0 2.6 2.2 1.8

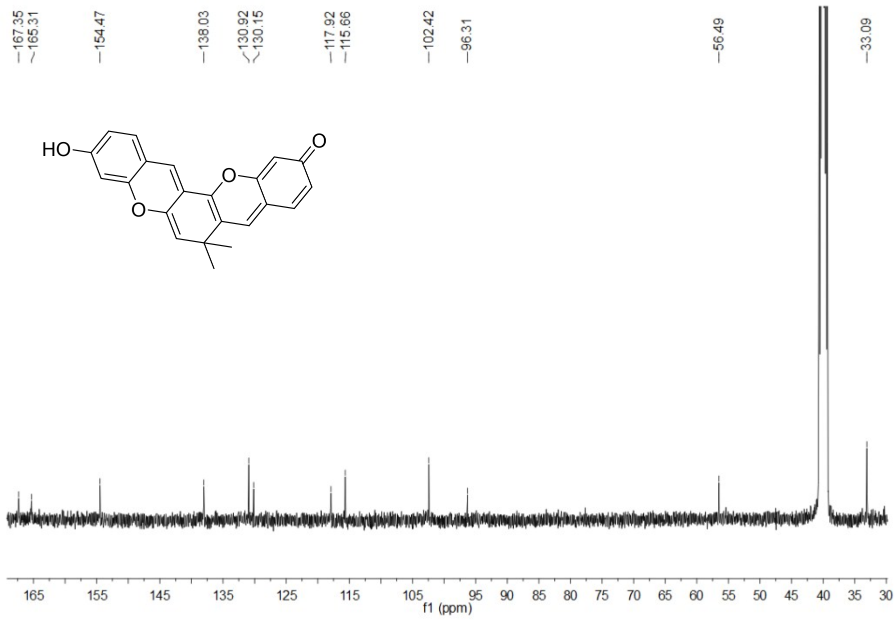

167.35
165.31
154.47
138.03
130.92
130.15
117.92
115.66
102.42
96.31
56.49
33.09
HO
O
165 155 145 135 125 115 105 95 90 85 80 75 70 65 60 55 50 45 40 35 30
f1 (ppm)

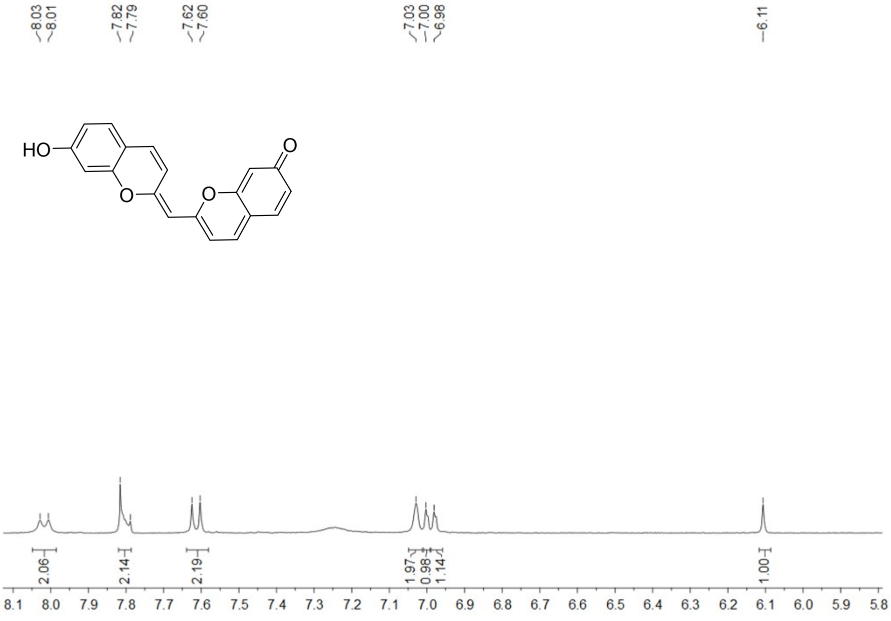
8.03
8.01
7.82
7.79
7.62
7.60
7.03
7.00
6.98
6.11
HO
O
O
O
2.06
2.14
2.19
1.97
0.98
1.14
1.00

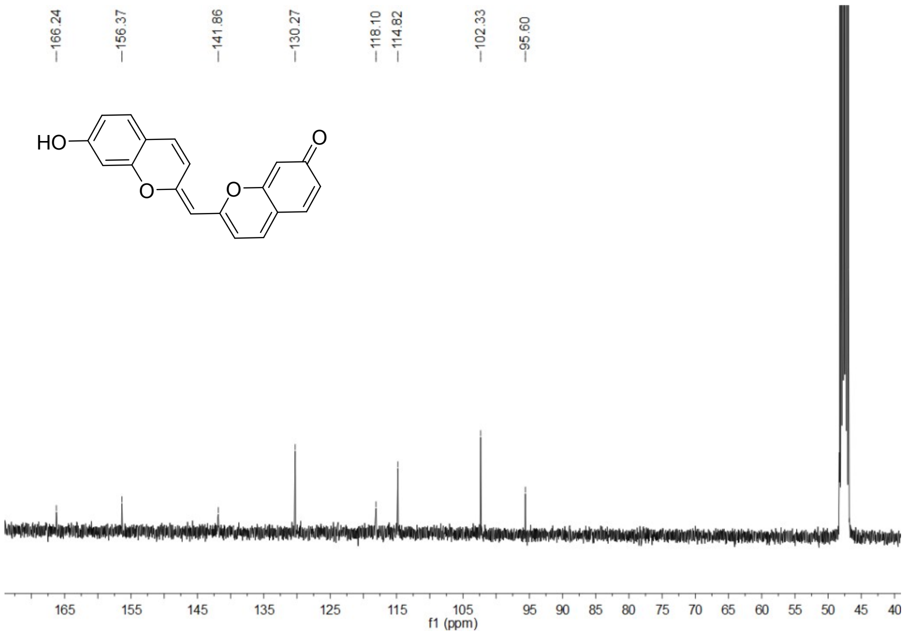
166.24
156.37
141.86
130.27
118.10
114.82
102.33
95.60
HO
O
O
O
f1 (ppm)

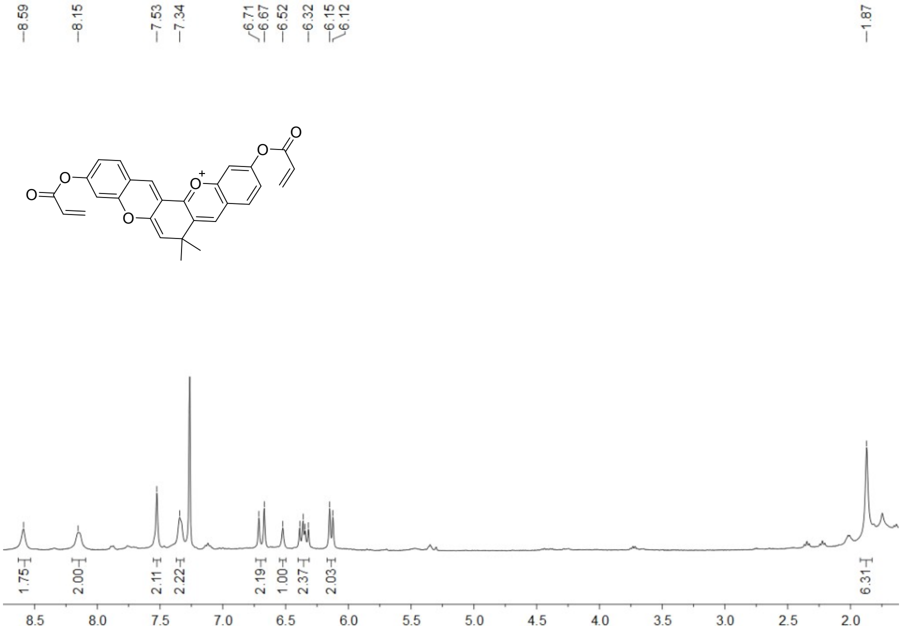

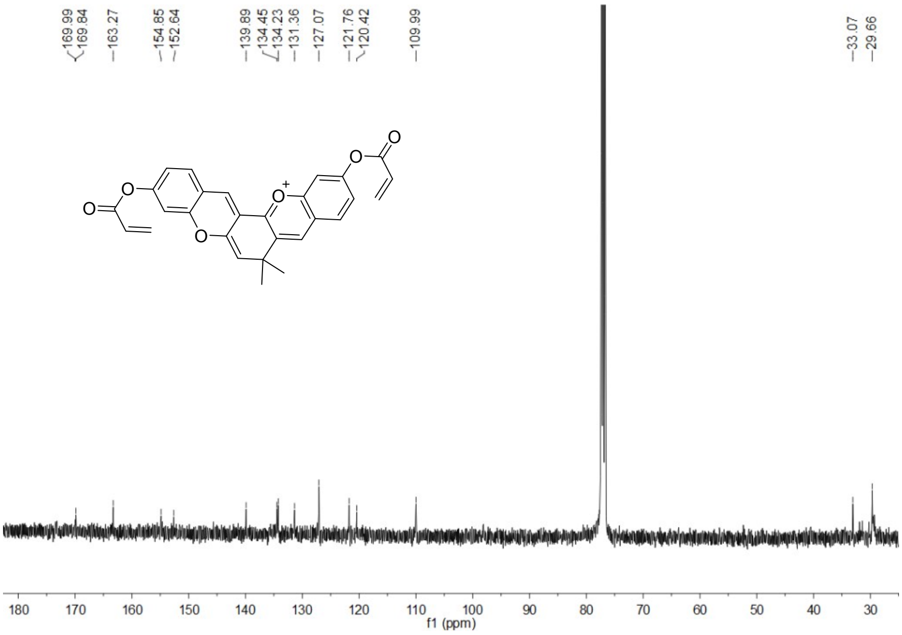

(1) Oushiki, D.; Kojima, H.; Terai, T.; Arita, M.; Hanaoka, K.; Urano, Y.; Nagano. T. *J. Am. Chem. Soc.*, 2010, **132**, 2795-2801.

(2) Kubin, R. F.; Fletcher, A. N. *J. Lumin.* **1982**, *27*, 455-462.
